# Cost-benefit analysis of interventions to protect care home residents in England against heat risks

**DOI:** 10.1088/2752-5309/ae25c8

**Published:** 2025-12-11

**Authors:** Andrew Ibbetson, John Cairns, Eleni Oikonomou, Giorgos Petrou, Anna Mavrogianni, Alastair Howard, Rajat Gupta, Mike Davies, Ai Milojevic

**Affiliations:** 1 Institute for Environmental Design and Engineering, The Bartlett School of Environment, Energy and Resources, University College London, London, United Kingdom; 2 Department of Public Health, Environments and Society, Faculty of Public Health and Policy, London School of Hygiene & Tropical Medicine, London, United Kingdom; 3 Department of Health Services Research and Policy, Faculty of Public Health and Policy, London School of Hygiene & Tropical Medicine, London, United Kingdom; 4 Low Carbon Building Research Group, School of Architecture, Oxford Brookes University, Oxford, United Kingdom

**Keywords:** care homes, heat mortality, adaptation, health modelling, climate change, cost-benefit

## Abstract

We examine the cost-benefit of selected physical and behavioural interventions to reduce indoor temperatures and associated health risks in UK care homes during periods of hot weather typical of the projected future climate. Cases of heat-related mortality under selected temperature scenarios were modelled for three care home settings. Published temperature-mortality functions for care home residents of England and Wales were applied to life tables for care home populations. Building physics modelling was used to assess the effect of interventions on summer indoor temperatures and associated mortality risks. The monetised value of quality-adjusted life years gained was assessed in relation to the capital, energy and maintenance costs of each intervention over its lifespan under a range of assumptions. Sensitivity analyses were used to characterise model uncertainty. Under the assumption that those who die of heat have the life expectancy of an average care home resident, we found evidence for cost-effective interventions. The cost-effectiveness of interventions varied across care homes, depending on each building’s specific characteristics. In a large, highly insulated care home with low thermal mass, increased thermal mass combined with window and door opening rules was the most cost-effective intervention; in a small care home with limited insulation and high thermal mass, active cooling in lounges using portable air-conditioners was most cost-effective; and in a medium sized care home with moderate insulation and thermal mass, shading combined with window and door opening rules was most cost-effective. Our results show that several interventions have the potential to be cost-effective in reducing heat-related risks to residents across a range of care home types. They also demonstrate that behavioural interventions, such as window and door opening, may be equally effective as physical interventions at reducing exposure to heat-related risks, provided they are fully adhered to.

## 1. Introduction

Residents of care homes are among the most vulnerable to the effects of heat (Arbuthnott and Hajat 2017, UKHSA 2025). In the UK, guidance for care home managers on how to protect residents from heat risks is currently limited to behavioural interventions, such as window and door opening (UKHSA 2024). This is despite evidence suggesting physical adaptations are effective in reducing heat risks in UK buildings, including care homes (Coley and Kershaw 2010, Gupta and Gregg 2012, Mavrogianni *et al* 2012, Porritt *et al* 2012, Oikonomou *et al* 2020, 2025). These physical adaptations include shading, increased thermal mass and active cooling.

Decisions to adopt interventions would ideally be informed by cost-benefit analysis, but there is currently limited evidence of their cost-effectiveness in care homes. Previous research by Ibbetson *et al* (2021) has demonstrated the challenge of assessing their cost-effectiveness due to uncertainties over loss of life expectancy from heat. Their study estimated that, under conservative life expectancy assumptions, the monetised value of life years gained from reducing indoor temperatures by around 1 °C during a hot summer could be justified in conventional cost-benefit terms for interventions costing around £800 per care home resident. However, a full cost-benefit analysis of candidate interventions across care homes of different building design has not been explored.

Improved guidance for care home managers on suitable interventions to protect care home residents against heat risk is becoming more important in the UK for two reasons. First, the share of care home residents is expected to rise due to population ageing. In 2024, 19% of the population was aged over 65, and this is projected to increase to 25% by 2060 (ONS 2024). Second, the increasing frequency and intensity of heatwaves due to climate change is likely to raise the exposure of care home residents to hot weather (Kendon *et al* 2018, 2022).

In this paper, we evaluate the cost-effectiveness of several interventions to reduce heat risks in care homes in England for impact on heat-related mortality. We assess cost-effectiveness by comparing the monetised value of quality-adjusted life years (QALYs) gained from the intervention against the associated capital, energy, and maintenance costs. To evaluate model uncertainty, we use sensitivity analyses. By selecting a set of deliberately contrasting interventions, from physical to behavioural adaptations, we compare differing approaches to heat protection rather than subtle variants of a similar approach. The cost-effectiveness of interventions is likely to vary depending on the characteristics of the care home under consideration. Therefore, we implement the interventions in three care homes of different building design to capture this variability.

The work presented here is the second of two companion papers. The first (Oikonomou *et al* 2025), analysed the physical performance of heat interventions in care homes, while the present paper extends the analysis to evaluate their cost-effectiveness.

## 2. Methods

### 2.1. Case study care homes

Our analyses were based on results for three care homes of contrasting building design and size, summarised in table 1. These care homes were chosen from the five care homes recruited for the original ClimaCare ‘pilot’ project (ClimaCare: Climate Resilience of Care Settings, 2019–20) to allow comparison of baseline heat risk for care homes of different building structure and form (Davies *et al* 2024). Of the three, care home CS1 is the largest and most modern care home. Built in 2013, it is a 5-storey purpose-built home with a capacity of 115 residents. It is a block and beam building with high levels of insulation, low thermal mass and a flat roof. Care home CS2 is the smallest care home, originally built in 1348 and converted in 2004, it is a 2-storey stone building capable of housing 11 residents. The building is uninsulated except for the loft, has a high thermal mass and a pitched roof. The characteristics of care home CS3 sit between the extremes of CS1 and CS2. Originally built in the 1980s and converted in 1993, it is a 3-storey brick building that can house 40 residents. It has moderate insulation and thermal mass, and a roof that is part flat and part pitched.

**Table 1.** Characteristics of the case study care homes.

*Code*	*Description*	*Build year*	*Residents (maximum)*	*Construction materials (estimated U-values*^*^a^*^ *in brackets)*		
				*Roof*	*Floor*	*Walls and windows* ^*^b^*^
CS1	5-storey purpose-built, modern block and beam building, highly insulated, low thermal mass, flat roof	2013	115 (115)	Block and beam, outmost layer insulation (0.1 W m^−2^ K)	Concrete, innermost layer insulation (0.2 W m^−2^ K)	Concrete block, innermost layer insulation (0.2 W m^−2^ K) Internal: plasterboard Double glazing (1.4 W m^−2^ K)

CS2	2-storey converted, stone building, uninsulated (except for loft), high thermal mass, pitched roof	1348 (2004 conversion)	8 (11)	Joist insulation (0.5 W m^−2^ K)	Solid, floorboards and covering, uninsulated (1.2 W m^−2^ K)	Stone wall, uninsulated (2 W m^−2^ K) Internal: brick Single glazing (4.7 W m^−2^ K)

CS3	3-storey converted, brick building of moderate insulation and thermal mass, part flat and part pitched roof (occupied in part)	1980s (1993 conversion)	38 (40)	Joist insulation (unoccupied pitched roof) rafter insulation (occupied, pitched roof), outermost layer insulation (flat roof)(0.6 W m^−2^ K)	Solid, uninsulated (1.2 W m^−2^ K)	Brick, cavity wall insulation (0.4 W m^−2^ K) Internal: brick Double glazing (3 W m^−2^ K)

a A measure of thermal transmittance (the rate of transfer of heat through matter) with SI units of watts per square metre-kelvin (W m^−2^ K).

b CS1 and CS3 had windows fitted with trickle vents. However, their operation and usage patterns throughout the year could not be verified. Therefore, their airflow contribution was not represented separately in our building physics models.

These care homes serve residents with both residential and nursing care needs, who are either bedbound or active. Bedbound residents spend most of their time in bedrooms and are confined to a bed or chair, whereas active residents are independently mobile and spend their time between bedrooms and lounges. Accordingly, we base the temperature exposure of bedbound residents on bedroom temperatures, while the exposure of active residents is based on averaging daytime lounge and night-time bedroom temperatures. Given the lack of readily available estimates of the proportion of bedbound residents in care homes, we use Census data on the general health of care home residents as a proxy (ONS 2023c), assuming that residents who self-report with ‘very bad’ health are bedbound. The 2021 Census likely underestimates the typical number of care home residents in ‘very bad’ health due to the impact of the COVID-19 pandemic. To account for this, we average the Census 2021 (8.8%) and Census 2011 (11.2%) estimates and assume that 10% of residents are bedbound and 90% are active. We explore the effect of this assumption on our results as part of our sensitivity analysis.

We assume that the care homes are always fully occupied. While national data suggests an occupancy of around 90% (DHSC 2024), care homes probably require high levels of occupancy to be economically sustainable, and so there are likely to be few with large numbers of empty beds. Instead, we expect that beds are unoccupied transiently due to turnover of residents, but this would have a negligible impact on our results. Our assumption of full occupancy is probably weakest for small care homes, where an empty bed would have a greater, negative effect on intervention cost-effectiveness compared to a large care home. Although for interventions such as air-conditioning, which could be switched off in unoccupied rooms, energy costs may decrease with lower occupancy, thereby reducing this effect.

### 2.2. Comparator

In each care home, we assessed interventions using a comparator scenario where the only cooling measure implemented was window opening during the daytime when temperatures were above 22 °C in occupied rooms. This aligns with the suggested window opening controls in the Chartered Institution of Building Services Engineers (CIBSE) guidelines for the assessment of overheating risks (CIBSE 2017). In practice, care homes may implement additional cooling measures, such as night ventilation. However, the prevalence of such practices in care homes is unknown and therefore we excluded them from our comparator. Thus, our results may reflect the maximal potential effect of the interventions studied.

### 2.3. Interventions

We assessed deliberately contrasting interventions that had been pre-screened for those offering the highest benefits against overheating (Oikonomou *et al* 2025), these are summarised in table 2. The interventions we tested were: shading with solar control window film (A1) or with external louvres and side fins (A2); increased thermal mass by stripping existing dry lining (B1) or by use of phase change materials (B2); active cooling by air conditioning in communal spaces only (C1) or in communal spaces and bedrooms (C2) which was either portable (CXa) or wall-mounted (CXb); night time ventilation applied in communal spaces only (D1) or in communal spaces and bedrooms (D3+); combinations of shading and night ventilation (A1D1, A2D1, A1A2D1); and combinations of increased thermal mass and night ventilation (B1D3+, B2D3+). To ensure that the codes assigned to our interventions are consistent with related publications (Oikonomou *et al* 2025), we have not altered them for this paper.

**Table 2.** Description of interventions. *T*_out_ = outdoor temperature; *T*_in_ = indoor temperature.

*Code*	*Intervention*	*Description*
A1	Shading: solar control window film	Solar window film application on the internal pane of existing windows, offering high protection from solar heat while allowing high transmission of natural light that is important for care home occupants.
A2	Shading: louvres and side fins	Louvres and side fins of 0.5 m projection applied according to the shading needs of each window, depending on orientation, overshading etc.
B1	Thermal mass: exposing existing high thermal mass materials	Existing thermal mass exposed by stripping dry lining in front of heavyweight materials where present.
B2	Thermal mass: use of phase change materials (PCM) panels	Where no high heavyweight materials, thermal mass added in the form of PCM panels.
C1(a/b)	Active cooling: air-conditioning to selected communal spaces	Cooled supply air in lounges using (a) portable or (b) wall-mounted air-conditioners to keep temperature below 26 °C. Windows/doors closed while the designated rooms are cooled.
C2(a/b)	Active cooling: air-conditioning to selected communal spaces and bedrooms	Cooled supply air in lounges using (a) portable or (b) wall-mounted air-conditioners to keep temperature below 26 °C. Windows/doors closed while the designated rooms are cooled.
D1	Behavioural: window/door rules on key apertures	Night ventilation applied to key areas. Internal doors between lounges and corridors remain open at nighttime.
D3+	Behavioural: window/door rules on key apertures and bedrooms	Night ventilation applied to key areas and bedrooms. Internal doors between lounges, corridors and bedrooms remain open at nighttime. Windows open only when *T*_out_ < *T*_in_.
A1A2D1	Combined: A1 + A2 + D1	Combination of interventions.
A2D1	Combined: A2 + D1	Combination of interventions.
A1D1	Combined: A1 + D1	Combination of interventions.
B1D3+	Combined: B1 + D3+	Combination of interventions.
B2D3+	Combined: B2 + D3+	Combination of interventions.

We assumed that behavioural interventions (D1 and D3+) were fully adhered to. However, this is unlikely to be the case due to common difficulties in sustaining behaviour change (Kelly and Barker 2016). Of our two behavioural interventions, D3+ is most likely to experience nonadherence, since it involves: (a) leaving doors between corridors and bedrooms open at nighttime and (b) opening windows only when the outdoor temperature is less than the indoor temperature. The former may not be supported by residents due to concerns over noise and security, while the latter would require knowledge of indoor and outdoor temperatures which is unlikely to be available. Thus, our results for these behavioural interventions are likely to overestimate their effectiveness.

For air-conditioning, we chose a setpoint of 26 °C to align with UKHSA guidance that advises temperatures in care homes are kept below this threshold to protect vulnerable residents (UKHSA 2024). Ideally, our choice would be informed by evidence on the typical setpoint used in care homes with air-conditioning, however, this was not available.

Some of our interventions may not be feasible in all care home settings. Therefore, we compare the cost-effectiveness of interventions using: (i) a fully incremental approach and (ii) a pair-wise comparison with a common comparator approach. A fully incremental approach is typically used when comparing more than two interventions (Paulden 2020). It aims to find the most cost-effective intervention by comparing the relative effectiveness and cost between interventions. For example, interventions that cost more and produce less health benefit than another intervention are said to be ‘dominated’ and ruled out. By contrast, the pair-wise comparison with a common comparator approach compares the effectiveness and cost of an intervention to a common comparator. Interventions are only ruled out if their incremental cost-effectiveness ratio (ICER) is more than the willingness-to-pay threshold. By using both approaches, we ensure that dominated interventions in our fully incremental approach are not overlooked as potential cost-effective options. For instance, an intervention with an ICER that is less than the willingness-to-pay-threshold may be ruled out in our fully incremental approach, but if it is the only feasible intervention in a care home it would be considered cost-effective.

### 2.4. Costs

We estimated the capital and maintenance costs of each intervention using quotes sourced from multiple suppliers in 2024. These were supplemented with an in-house quotation to serve as a baseline, enabling comparative analysis and ensuring the validity of cost estimates. We selected care home CS3 as a costing test base, since its characteristics in terms of size, age, and thermal properties were closer to the average for the three care homes in this study. The resulting costing framework was then extrapolated by window, wall or floor area (depending on the intervention) to estimate costs for the other two care homes. This extrapolation method assumes that there are no purchasing economies of scale.

We derived quote averages from a comprehensive work description, which included a bill of quantities (BOQs), architectural drawings, photographs, and detailed information about the existing conditions of the rooms and building elements in care home CS3. The BOQ also included provisional sums for electrical and builders’ work, as the full scope of these work items was unknown. It also accounted for preliminaries, including site storage, staff welfare facilities, health and safety provisions, and a 10% risk allowance. We only considered quotes to be valid if they included a comprehensive breakdown of costs, ensuring transparency and reliability. This approach gave two or three valid cost estimates per intervention, except for intervention B1 (stripping existing dry lining) as no contractors provided a quote. Hence, we used an in-house cost estimate for B1. The contractors provided estimates for the expected lifespan of each intervention, which we validated against publicly available data (table 3). Intervention B1 has an unlimited lifespan, but its actual lifespan is likely to be limited by that of the care home, therefore we assume a lifespan of 50 years. We examine the effect of intervention lifespans on our results as part of our sensitivity analysis.

**Table 3.** Model parameters. Interventions: solar control window film (A1) or with external louvres and side fins (A2); increased thermal mass by stripping existing dry lining (B1) or by use of phase change materials (B2); active cooling by air conditioning in communal spaces only (C1) or in communal spaces and bedrooms (C2). Weather files: DSY = design summer year; TRY = test reference year.

*Parameter*		*Value*	*Source*	*Distribution*
England and Wales care home annual mortality rate (per person)				

Male	Age 65–69	0.122	ONS (2023b)	Not varied in sensitivity analysis
	Age 70–74	0.191		
	Age 75–79	0.253		
	Age 80–84	0.316		
	Age 85–89	0.376		
	Age 90+	0.448		
Female	Age 65–69	0.116		
	Age 70–74	0.157		
	Age 75–79	0.190		
	Age 80–84	0.227		
	Age 85–89	0.264		
	Age 90+	0.341		

England and Wales care home annual deaths				

Male	Age 65–69	710	ONS (2023b)	Not varied in sensitivity analysis
	Age 70–74	1725		
	Age 75–79	2860		
	Age 80–84	4430		
	Age 85–89	5590		
	Age 90+	6720		
Female	Age 65–69	590		
	Age 70–74	1535		
	Age 75–79	3210		
	Age 80–84	6570		
	Age 85–89	11 195		
	Age 90+	22 630		

EQ5D norms				
Age 65–74		0.773	Janssen and Szende (2014)	Not varied in sensitivity analysis
Age 75+		0.703		
Weather file frequency of occurrence (%)			Eames (2016) and expert opinion—see Methods 2.3 for details	Uniform for TRY: min = 0.8*central estimate; max = 1.2*central estimate. Conditional on TRY value, we uniformly reweight DSY frequencies to ensure frequencies sum to 100%.
DSY1		16		
DSY2		8		
DSY3		4		
TRY		72		

Heat temperature threshold (*T*_h_) (°C)		17	Hajat *et al* (2007)	Uniform: min = 16; max = 18

Temperature-mortality gradient: RR per °C		1.05	Hajat *et al* (2007)	Uniform: min = 1.03; max = 1.07

Summer mortality rate adjustment		0.876	ONS (2023d)	Normal: SE = 0.001

Discount rate (%)		3.5	NICE (2022)	Not varied in sensitivity analysis

Boiler energy efficiency (%)		90	DECC (2018)	Uniform: min = 85; max = 95

Air-conditioning energy efficiency (%)				
Wall-mounted (split system)		450	European Union (2012)	Uniform: min = 0.8*central estimate; max = 1.2*central estimate
Portable		250	EU (2012)	

Energy cost per kWh (£)				
Electricity (standard)		0.245	Energy Saving Trust (2025)	Uniform: min = 0.8*central estimate; max = 1.2*central estimate
Gas		0.062		

Care home occupancy (%)		100	Assumption—see Methods 2.1	Not varied in sensitivity analysis

Bedbound residents (%)		10	ONS (2023c)	Uniform: min = 0; max = 20

Time horizon (years)		1		Not varied in sensitivity analysis

Intervention costs		Care home and weather dependent. See figures A8–A9 and Methods 2.4 for details		Uniform: min = 0.8*central estimate; max = 1.2*central estimate

Intervention lifespan (years)				
A1		25	Quotation—see Methods 2.4	Uniform: min = 0.8*central estimate; max = 1.2*central estimate
A2		30	Quotation—see Methods 2.4	
B1		50	Assumption—see Methods 2.4	
B2		30	Kharbouch (2022)	
C1		15	Quotation—see Methods 2.4	
C2		15	Quotation—see Methods 2.4	

We annualised capital costs by dividing them by an annualisation factor (Walker 2002), ${\text{AF}}$, given by

\begin{equation*}{\text{A}}{{\text{F}}_{\text{i}}} = { }\frac{{{{\left( {1 + d} \right)}^{{n_{\text{i}}}}} - 1}}{{d{{\left( {1 + d} \right)}^{{n_{\text{i}}}}}}},\end{equation*}

where, $d$ is the discount rate and ${n_{\text{i}}}$ is the lifespan of intervention $i$. We used a discount rate of 3.5% per annum in line with the latest UK National Institute for Health and Care Excellence (NICE) guidance (2022).

We estimated the operational heating and cooling energy costs of the care homes under each intervention using building physics simulations. To derive costs, idealised heating energy use was multiplied by an estimate of the unit cost of gas (EST 2025) and divided by the boiler energy efficiency (DECC 2018). Similarly, cooling energy use was multiplied by an estimate of the unit cost of electricity (EST 2025) and divided by the air-conditioning energy efficiency (EU 2012). The estimated unit costs of gas and electricity correspond to average estimates for the year in which they were accessed. Therefore, they were not based on projections, however, we evaluated the effect of changes in gas and electricity prices on our results as part of our sensitivity analysis.

Our interventions included behavioural adaptations, such as window opening, whose costs were not easily quantified and were assumed to be nil. We therefore excluded wholly behavioural interventions (D1 and D3+) from our cost-effectiveness analysis but included them in an assessment of net monetary benefit, to allow some degree of comparison across all interventions.

We report all costs in £ 2024 GBP.

### 2.5. Climate projection and weather data

**Figure A1.**
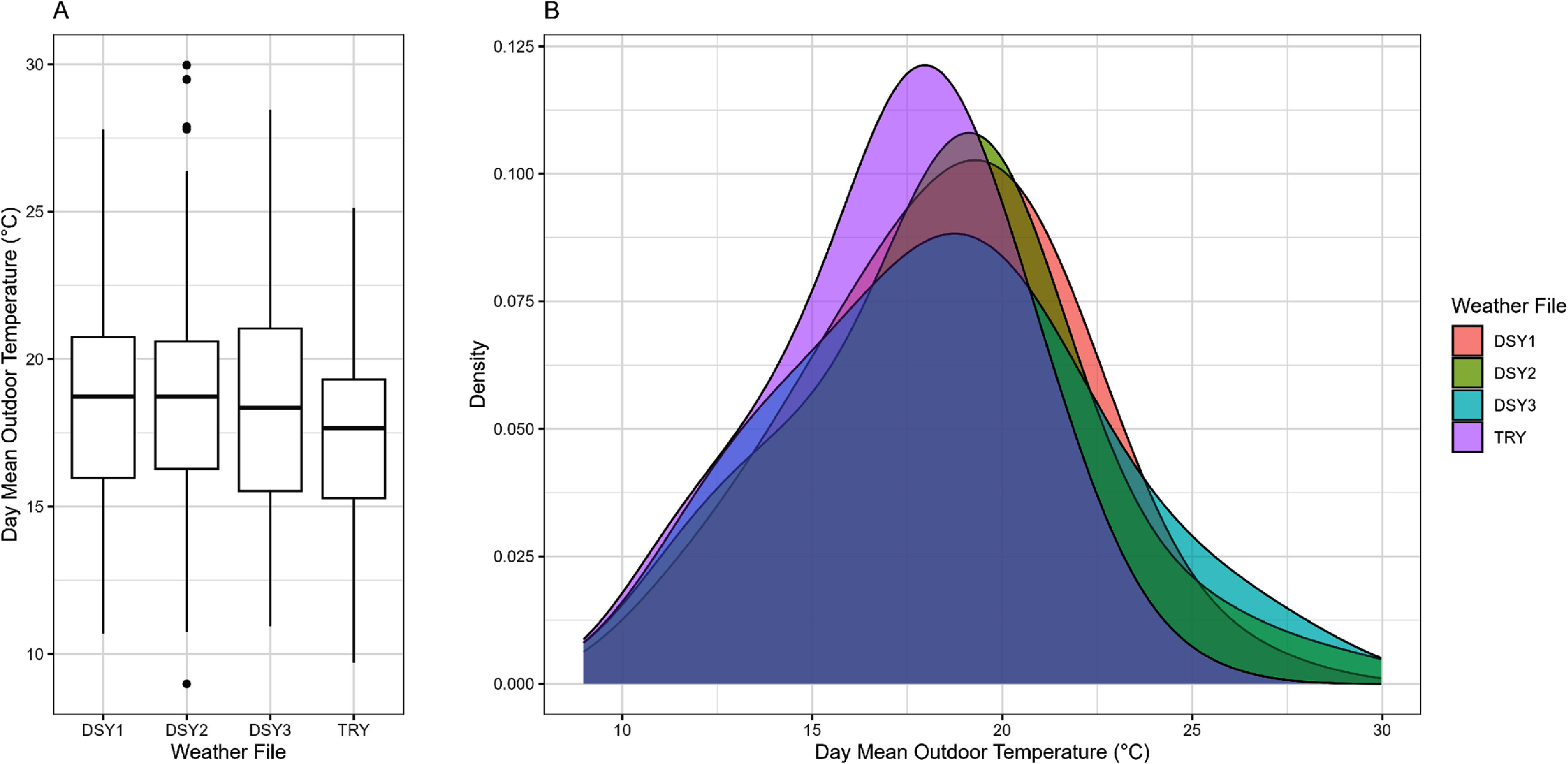
(A) Boxplot and (B) density plot of the day mean outdoor temperature by weather file. Weather files: DSY = design summer year; TRY = test reference year.

we assessed the effect of our interventions under UK Climate Projections 2009 (UKCP09) weather files developed by CIBSE. Although UK Climate Projections 2018 (UKCP18) provide the most up-to-date probabilistic climate projections, weather files based on these projections were not available at the time of our study. Thus, we selected UKCP09 weather files to closely align with the UKCP18 probabilistic data for 2 °C and 4 °C Global Mean Surface Temperature increases above pre-industrial levels. We used the ‘Design Summer Year’ (DSY1, DSY2 and DSY3) and ‘Test Reference Year’ (TRY) datasets published by CIBSE for London Heathrow under the 2020s high emissions, 50th percentile scenario (CIBSE, 2024). The DSY weather files are selected from meteorological records of the period 1977–2004, morphed according to UKCP09 projections using published methods. These methods involve ranking each year according to a set of metrics that capture different aspects of overheating, such as duration and intensity of heat. According to these rankings, three DSYs are then chosen to represent summers with different types of overheating risk. DSY1 represents a moderately warm summer, DSY2 represents a summer with a short, intense warm spell, and DSY3 represents a summer with a long, less intense warm spell. Whilst TRY files represent typical meteorological conditions derived from average monthly data for the same period. The temperature distribution for each weather file is shown in figure A1.

We combined the results from each weather file using return periods (*r*), a statistical measure of the frequency by which a future summer that is as warm or warmer than the weather file is expected to occur: *r*DSY1 = 6.7 years; *r*DSY2 = 15.5 years; *r*DSY3 = 23.7 years; *r*TRY = 2.0 years (Eames 2016). We assumed that these return periods do not change over our time horizon. Specifically, the weather associated with each weather file is assumed to occur with a frequency within a 25 year period as follows: DSY1 = 4/25; DSY2 = 2/25; DSY3 = 1/25; and TRY = 18/25 (correspondence with Eames).

### 2.6. Estimating heat-related mortality and loss of quality-adjusted life

Using data published by Hajat *et al* (2007), we estimated heat-related mortality by assuming a log-linear temperature-mortality function. While they did not specify this function exactly, their graphical presentation is in line with a relative risk of 1.05 for each degree Celsius increase in daily mean temperature above a temperature threshold of 17 °C based on a lag of 0–1 d. By applying population level estimates of relative risk to individual care homes, we assume that our study care homes represent the average care home in England and Wales. However, each care home is likely to have a unique, temperature-mortality relationship which is based on its building features and the characteristics of its population in terms of, for example, age and prevalence of comorbidities. We explore the effect of this uncertainty on our results by varying the heat threshold and temperature-mortality gradient in our sensitivity analyses.

For each day, $i$, we computed the relative risk, ${\text{R}}{{\text{R}}_{\text{i}}}$, for heat-related mortality as

\begin{equation*}{\text{R}}{{\text{R}}_{\text{i}}} = {\text{ RR}}_{{\text{coef}}}^{\left( {{T_{{\text{mean}}}}_{\text{i}} - {T_{\text{h}}}} \right)},\end{equation*}

where, ${T_{{\text{mean}}}}_{\text{i}}$ is the two-day (lag 0–1 d) mean outdoor temperature on day $i$; ${T_{\text{h}}}$ is the temperature threshold for heat-related mortality; and ${\text{R}}{{\text{R}}_{{\text{coef}}}}$ is the relative risk (1.05) for a one degree Celsius increase in temperature above ${T_{\text{h}}}$.

From this we derived the daily number of heat attributable deaths, ${\text{H}}{{\text{D}}_j}$, as

\begin{equation*}{\text{H}}{{\text{D}}_j} = { }\sum\limits_{\text{i}} \frac{{{\text{R}}{{\text{R}}_{\text{i}}} - 1}}{{{\text{R}}{{\text{R}}_{\text{i}}}}}*{D_j},\end{equation*}

where, ${D_j}$ is the daily season-average age- and sex-specific mortality rate for person $j$; the age- and sex-specific mortality rates were derived from estimates of age- and sex-specific care home mortality published by ONS (ONS 2023b) which we seasonally adjusted using ONS data on winter mortality in care homes (ONS 2023d).

We computed QALYs lost by multiplying the number of heat-related deaths by the average life expectancy and quality of life of care home residents. We used published age-specific quality of life estimates for England which were measured using EQ5D (Janssen and Szende 2014). These estimates are likely to overestimate the quality of life of care home residents who probably have reduced quality of life compared to the general population due to ill health (O’Neill *et al* 2020). However, there are a lack of reliable quality of life estimates for care home residents, who, due to their frailty, are often unable to provide self-reported measures (Usman *et al* 2019).

### 2.7. Assessing the effect of building adaptations on indoor temperature and heat-related mortality

We assessed the impact of our interventions on heat-related mortality using a similar approach previously applied by Taylor *et al* (2018) and Ibbetson *et al* (2021), where a change in daily indoor temperature caused by an intervention, will have an equivalent effect on mortality as a corresponding shift along the outdoor temperature-mortality function. Thus, if the intervention reduces the indoor temperature by 0.5 °C, we assume that the effective outdoor temperature for that individual is also reduced by 0.5 °C.

We modelled the effect of each intervention on the current and future overheating risks of the three case study care homes in DesignBuilder (DesignBuilder Software Ltd, 2024), an interface for EnergyPlus (U.S. DOE 2020). EnergyPlus is an energy analysis and thermal load simulation engine tested against the International Energy Agency Building Energy Simulation Test and Diagnostic Method building load and ANSI/American Society of Heating, Refrigerating and Air-Conditioning Engineers Standard 140. The models were validated against empirical data collected in the summer of 2019 (for more details see: Oikonomou *et al* 2025). The model produced hourly temperature data which we converted to two-day (lag 0–1 d) mean temperature for consistency with epidemiological estimates of the relative risk of heat-related mortality.

### 2.8. Time horizon, perspective and willingness-to-pay threshold

Our methodological assumptions imply that year-on-year there is no change in health benefit or cost for each intervention, and therefore we choose a time horizon of one year. Since outdoor temperatures rarely exceed the heat threshold outside the months May to September (two days in DSY2 only), our health calculations focus on this period. However, the operational costs of our interventions vary outside this period, as a result they are calculated over the full year.

The perspective of our analysis is that of a care home owner. For that reason, only costs attributable to the care home are included in our analysis. Whereas other costs, such as those incurred by the healthcare system, are excluded. Although care homes in England are nearly all privately owned (Blakeley and Quilter-Pinner 2019), an estimated 63% of residents are state funded (ONS 2023a). Therefore, future research may wish to consider taking a government perspective.

We assume a willingness-to-pay threshold of £20 000 per QALY in line with the latest NICE guidance (NICE 2022). This is different from our previous work which used a threshold of £30 000 per QALY (Ibbetson *et al* 2021). Our choice to use the lower threshold is a conservative decision informed by NICE guidance which states that interventions above this threshold may only be considered cost-effective depending on certain factors, such as uncertainty around the ICER and adequate assessment of changes in quality of life and health gain. The willingness-to-pay threshold recommended by NICE contrasts with the £70 000 per QALY threshold proposed by HM Treasury (2024).

### 2.9. Sensitivity analysis

**Figure A2.**
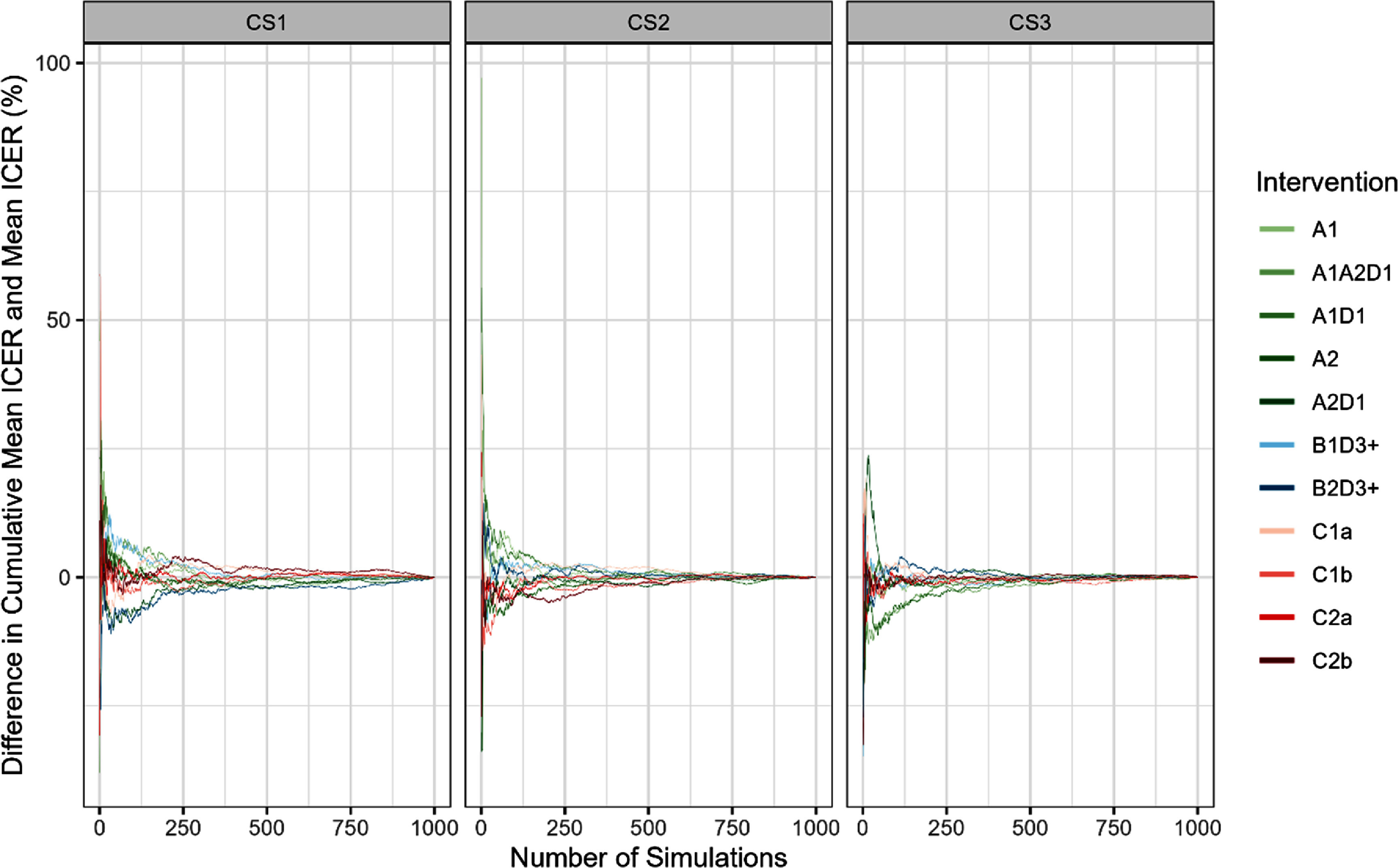
Probabilistic sensitivity analysis convergence by care home (CS1, CS2 and CS3) and intervention. Interventions: solar control window film (A1) or with external louvres and side fins (A2); increased thermal mass by stripping existing dry lining (B1) or by use of phase change materials (B2); active cooling by air conditioning (*a* = portable; *b* = wall-mounted) in communal spaces only (C1) or in communal spaces and bedrooms (C2); night time ventilation applied in communal spaces only (D1) or in communal spaces and bedrooms (D3+). ICER = incremental cost-effectiveness ratio.

To evaluate the robustness of our results to nonlinearities in our model, we conducted probabilistic sensitivity analysis with sampling from the probability distributions of several model parameters (table 3). Due to a lack of evidence to support specific distributions for most parameters, we conservatively assumed a uniform distribution with a wide range (in most cases ±20% of the central estimate). After testing for model convergence by visual inspection of trace plots (Hatswell *et al* 2018), we performed our analysis using 1000 simulations (figure A2). The results of our probabilistic sensitivity analysis are presented using the cost-effectiveness plane and cost-effectiveness acceptability curves (Barton *et al* 2008).

To evaluate the effect of individual changes in input parameter values on our cost-effectiveness estimates, we conducted a one-way deterministic sensitivity analysis. We selected high and low parameter values based on the maximum and minimum values (or 95% confidence interval where specified) of the parameters assigned distributions (table 3). We present the results of this analysis using tornado plots (Briggs *et al* 2012).

Due to uncertainty in the degree of life shortening experienced by those dying of heat (Armstrong *et al* 2017, Rehill *et al* 2015), we explored the effect of a conservative estimate of loss of life expectancy on our results. This conservative estimate assumes that those who die of heat are frail residents with an average life expectancy of six months, regardless of age.

## 3. Results

### 3.1. Effect of building interventions on temperature

**Figure A3.**
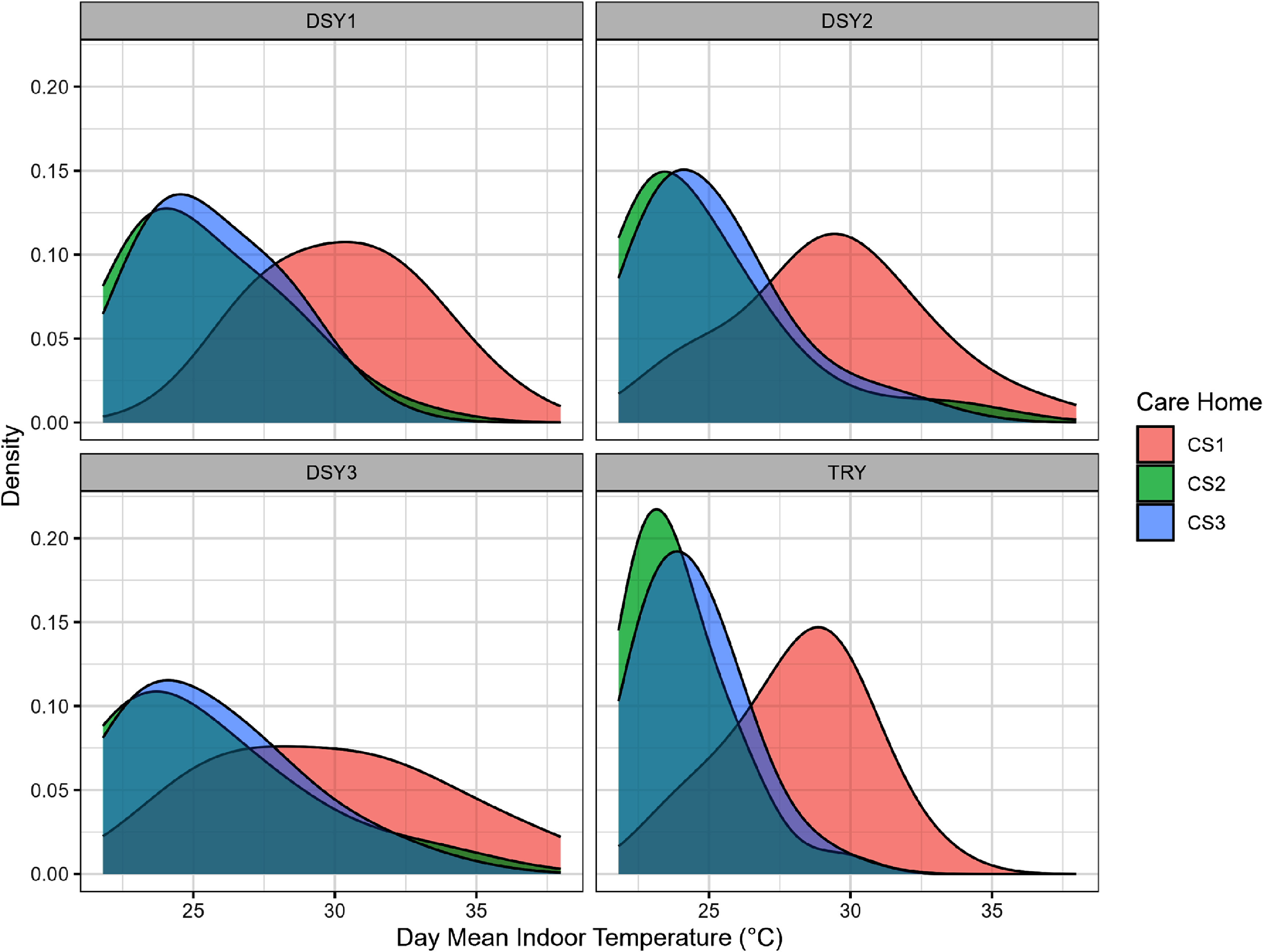
Distribution of day mean indoor temperature by care home and weather file (DSY1, DSY2, DSY3 and TRY) for the comparator. Weather files: DSY = design summer year; TRY = test reference year.

**Figure A4.**
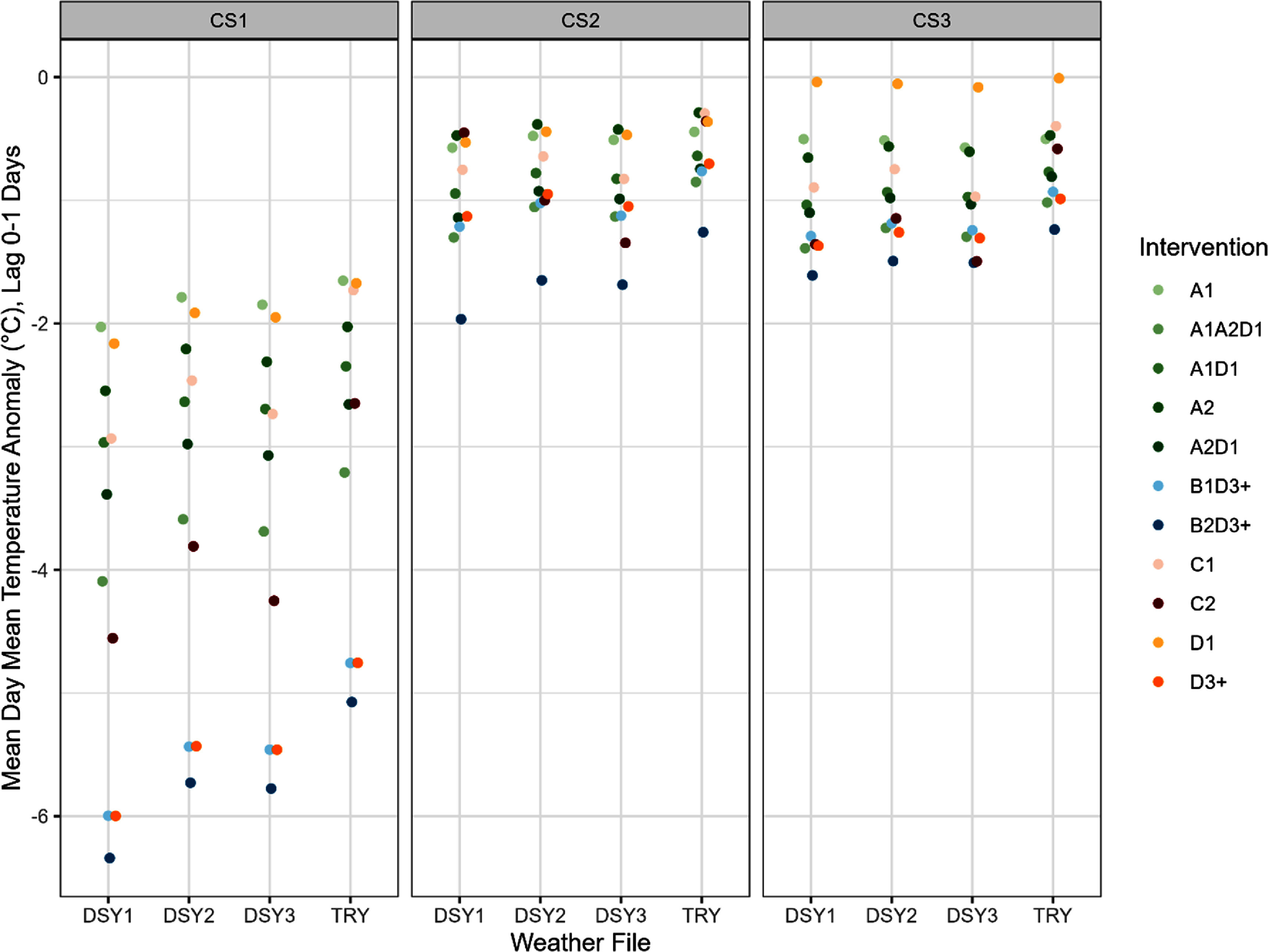
Mean day mean temperature anomaly, i.e. the deviation in daily mean indoor temperature, due to interventions by care home (CS1, CS2 and CS3), weather file and intervention. Interventions: solar control window film (A1) or with external louvres and side fins (A2); increased thermal mass by stripping existing dry lining (B1) or by use of phase change materials (B2); active cooling by air conditioning (*a* = portable; *b* = wall-mounted) in communal spaces only (C1) or in communal spaces and bedrooms (C2); night time ventilation applied in communal spaces only (D1) or in communal spaces and bedrooms (D3+). Weather files: DSY = design summer year; TRY = test reference year.

The interventions tested had the largest effect on temperature in care home CS1, which was also the warmest care home at baseline (figure A3). In CS1, mean temperature anomalies, i.e. the deviation in daily mean indoor temperature due to interventions, ranged from around −1.6 to −6.2 °C (figure A4). Whereas for care homes CS2 and CS3, anomalies ranged from −0.3 to −2 °C. In all care homes, the most negative average temperature anomalies were achieved by B2D3+.

**Figure A5.**
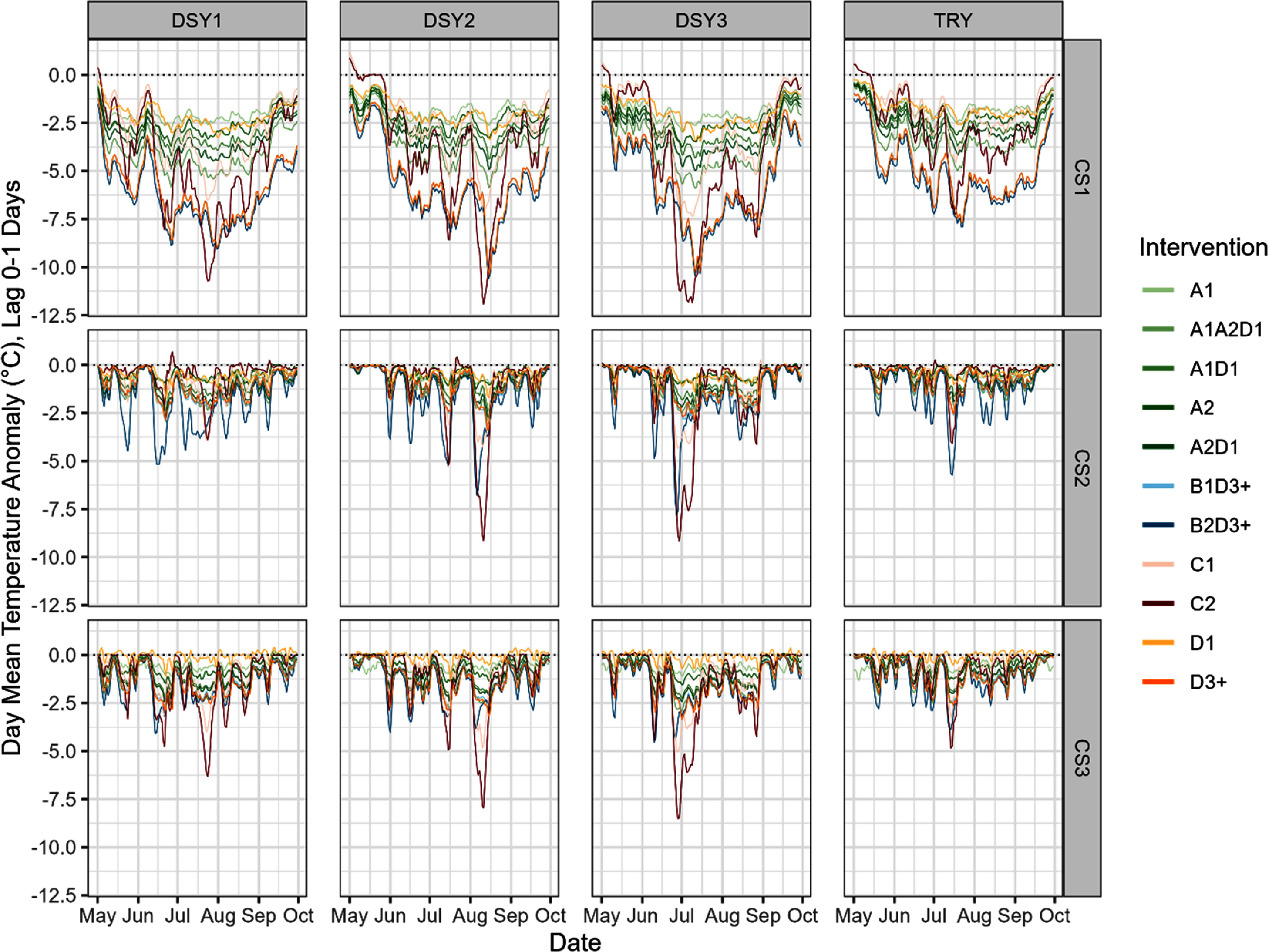
Day mean temperature anomaly, i.e. the deviation in daily mean indoor temperature, by care home (CS1, CS2 and CS3), weather file (DSY1, DSY2, DSY3 and TRY) and intervention. Interventions: solar control window film (A1) or with external louvres and side fins (A2); increased thermal mass by stripping existing dry lining (B1) or by use of phase change materials (B2); active cooling by air conditioning (a = portable; *b* = wall-mounted) in communal spaces only (C1) or in communal spaces and bedrooms (C2); night time ventilation applied in communal spaces only (D1) or in communal spaces and bedrooms (D3+). Weather files: DSY = design summer year; TRY = test reference year.

**Figure A6.**
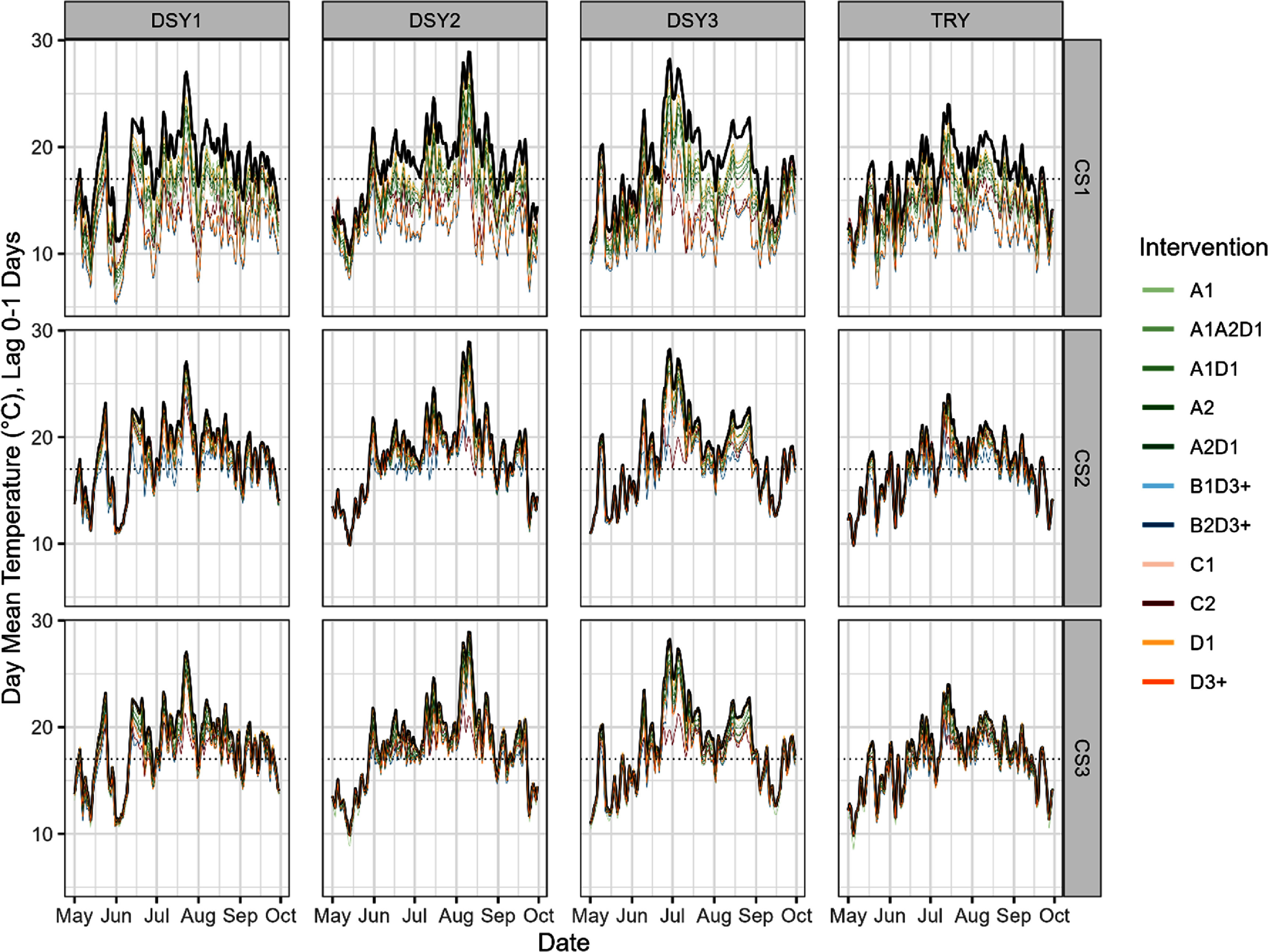
Day mean outdoor temperature (black line) and effective outdoor temperature after subtraction of the intervention indoor anomaly, i.e. the deviation in daily mean indoor temperature, by care home (CS1, CS2 and CS3) and weather file (DSY1, DSY2, DSY3 and TRY). Interventions: solar control window film (A1) or with external louvres and side fins (A2); increased thermal mass by stripping existing dry lining (B1) or by use of phase change materials (B2); active cooling by air conditioning (*a* = portable; *b* = wall-mounted) in communal spaces only (C1) or in communal spaces and bedrooms (C2); night time ventilation applied in communal spaces only (D1) or in communal spaces and bedrooms (D3+). Weather files: DSY = design summer year; TRY = test reference year.

On the hottest days, C2 performed best, reducing day mean indoor temperatures by up to 8 °C in all care homes (figures A5 and A6). On these days, B2D3+ performed almost as well as C2 in care homes CS1 and CS2, however, it was much less effective in care home CS3.

In CS1, several interventions consistently produced substantial temperature anomalies, reducing the effective day mean outdoor temperature to below the heat mortality threshold on most days, thereby removing all risk of heat-related death (figure A6). This occurred much less often in CS2 and CS3.

**Figure A7.**
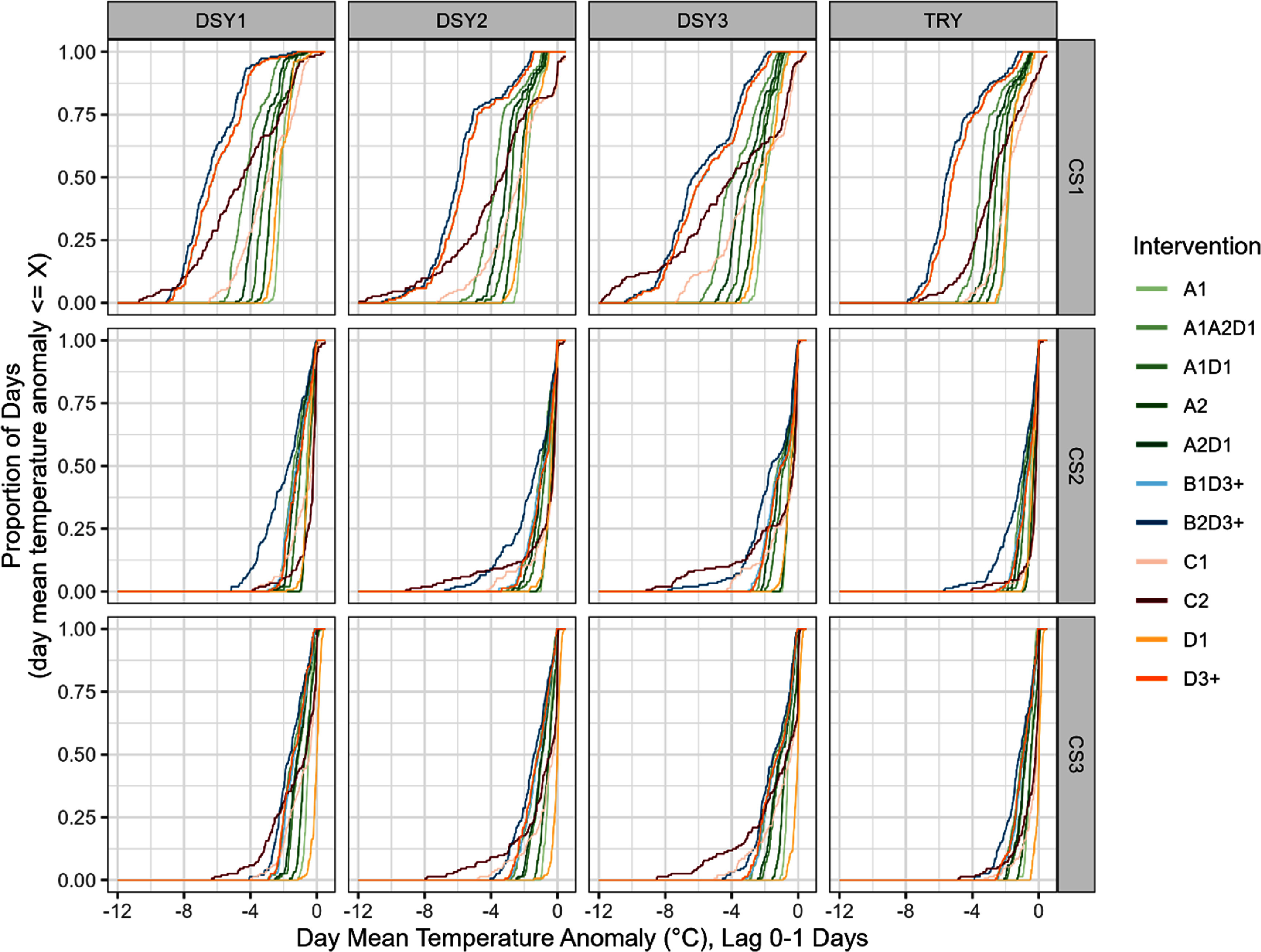
Cumulative distribution of day mean temperature anomaly, i.e. the deviation in daily mean indoor temperature, by case study care home (CS1, CS2 and CS3), weather file (DSY1, DSY2, DSY3 and TRY) and intervention. Interventions: solar control window film (A1) or with external louvres and side fins (A2); increased thermal mass by stripping existing dry lining (B1) or by use of phase change materials (B2); active cooling by air conditioning (*a* = portable; *b* = wall-mounted) in communal spaces only (C1) or in communal spaces and bedrooms (C2); night time ventilation applied in communal spaces only (D1) or in communal spaces and bedrooms (D3+). Weather files: DSY = design summer year; TRY = test reference year.

For most interventions the distribution of day mean temperature anomalies was normally distributed, except for C1, C2, D3+, B1D3+ and B2D3+ (figure A7). Temperature anomalies for C1 and C2 were typically left skewed with a long tail of large anomalies. In contrast, D3+, B1D3+ and B2D3+ produced anomalies which were right skewed in care home CS1 but left skewed in care homes CS2 and CS3.

### 3.2. Costs of building interventions

**Figure A8.**
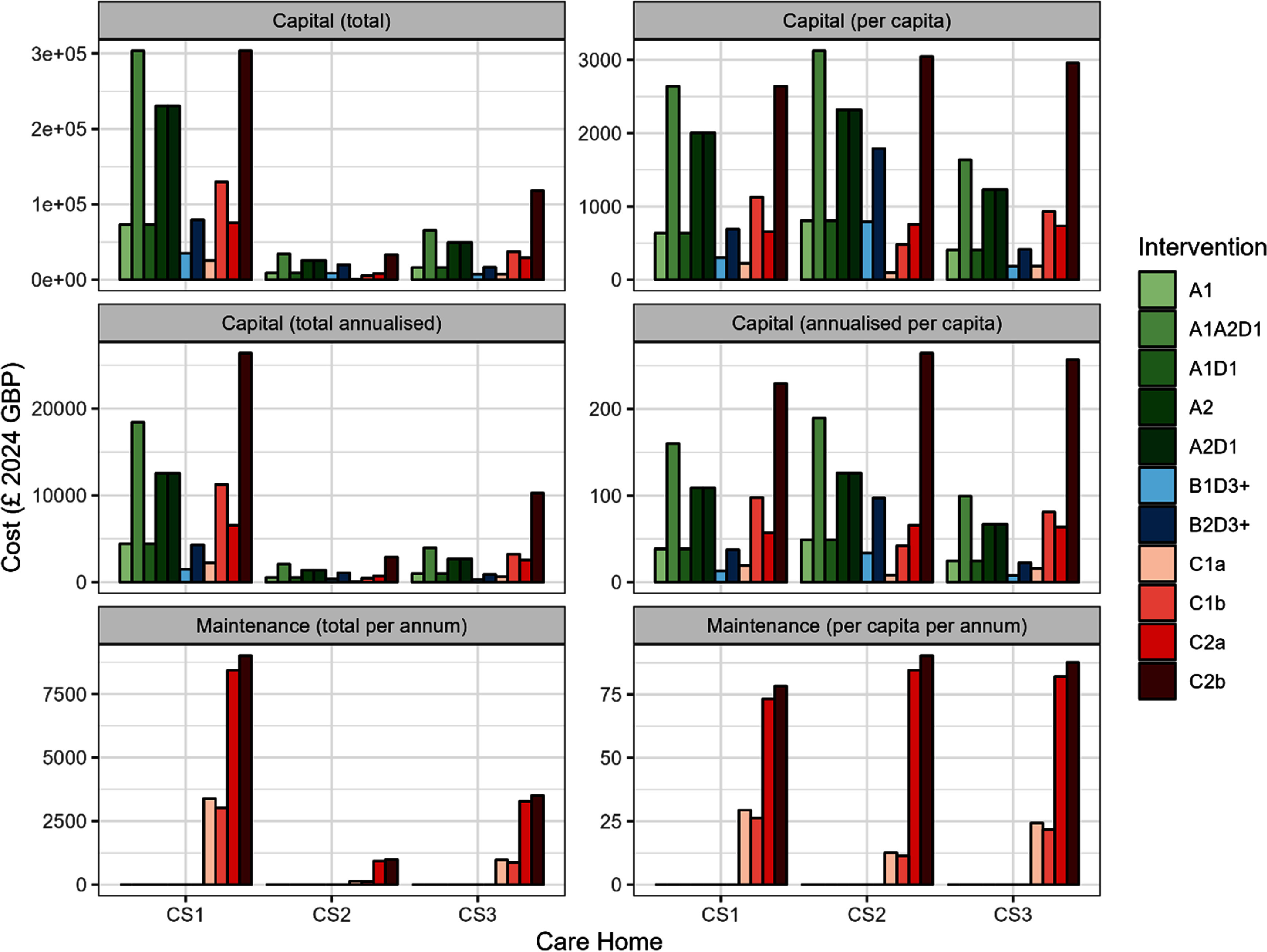
Intervention capital and maintenance costs by case study care home (CS1, CS2 and CS3) and intervention. Interventions: solar control window film (A1) or with external louvres and side fins (A2); increased thermal mass by stripping existing dry lining (B1) or by use of phase change materials (B2); active cooling by air conditioning (*a* = portable; *b* = wall-mounted) in communal spaces only (C1) or in communal spaces and bedrooms (C2).

For all care homes, C2b generally had the highest capital cost, with per capita costs of around £2600–3000 (figure A8). It was also the most expensive intervention on an annualised basis, with per capita costs of around £250 per annum. Maintenance costs were greatest for C2a and C2b, at around £75–85 per capita per annum, followed by C1a and C1b with costs around £12–30 per capita per annum. All other interventions had negligible maintenance costs.

Capital costs per capita were typically highest in CS2, followed by CS1 and CS3. Air-conditioning costs, which were largely determined by the number of communal areas per resident, were an exception to this trend.

**Figure A9.**
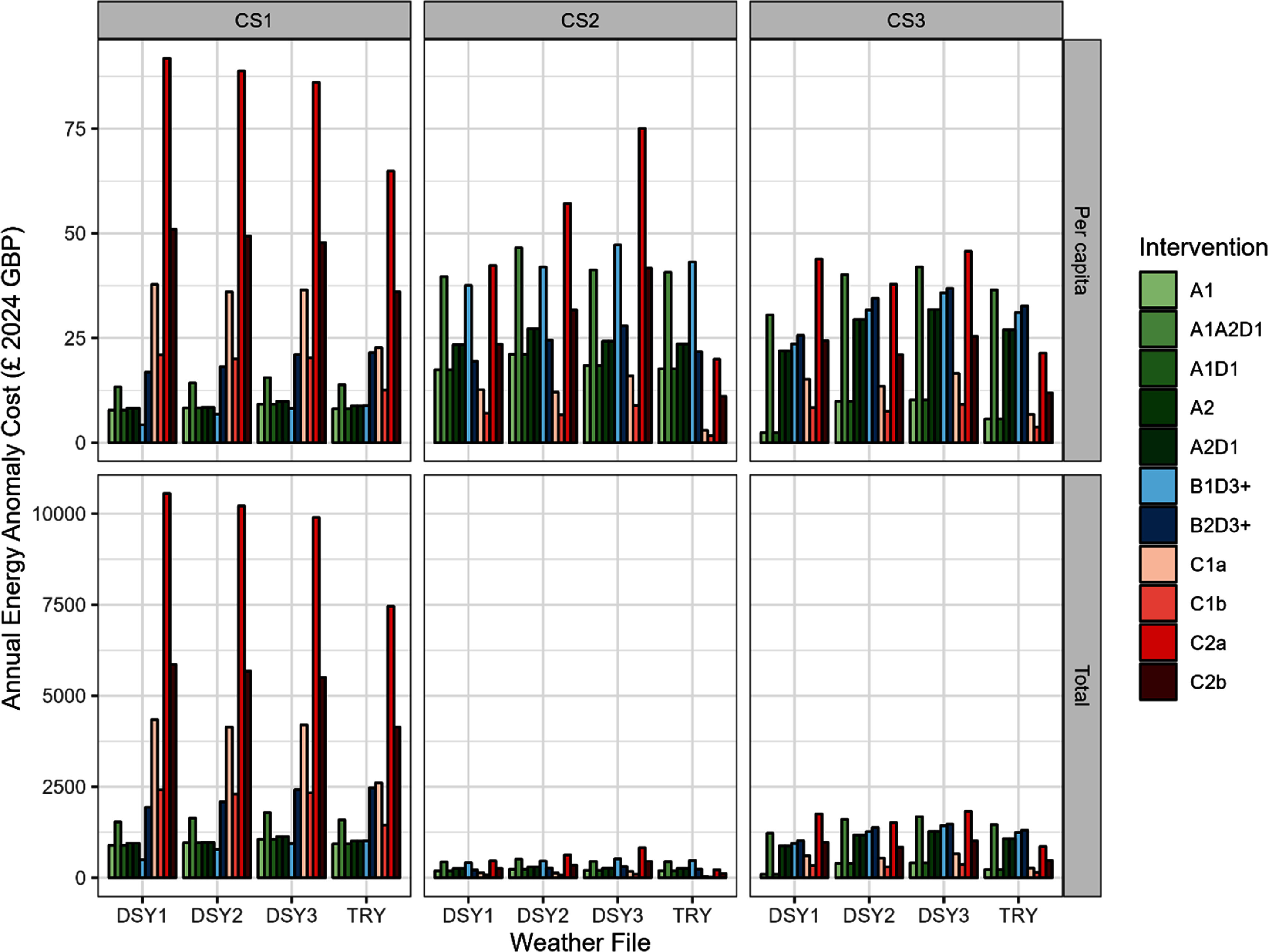
Total and per capita cost of the annual energy use anomaly by case study care home (CS1, CS2 and CS3), intervention and weather file. Interventions: solar control window film (A1) or with external louvres and side fins (A2); increased thermal mass by stripping existing dry lining (B1) or by use of phase change materials (B2); active cooling by air conditioning (*a* = portable; *b* = wall-mounted) in communal spaces only (C1) or in communal spaces and bedrooms (C2). Weather files: DSY = design summer year; TRY = test reference year.

Interventions increased the annual energy costs of care homes by £2–90 per capita (figure A9). Interventions producing the largest increase in per capita energy costs were, for CS1: C2a (£57–90); CS2: A1A2D1 and B1D3+ (£38–47); and CS3: A1A2D1, A2, A2D1, B1D3+ and B2D3+ (£22–42). For air-conditioning interventions, energy costs were greater in the warmer weather files, while there were no clear patterns in energy costs for the other interventions between weather files.

### 3.3. Effect of building interventions on health

**Figure A10.**
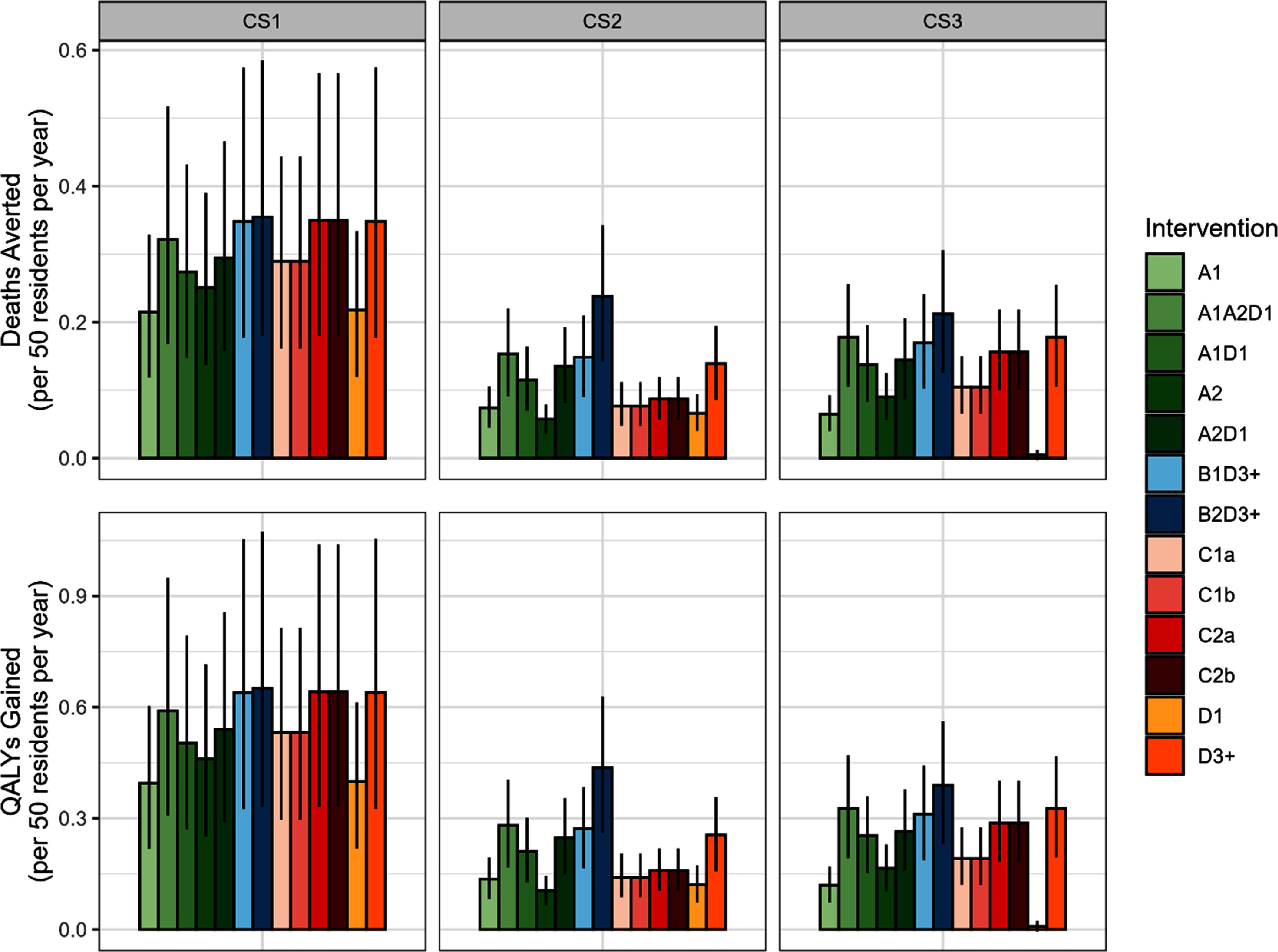
Deaths averted and quality-adjusted life years (QALYs) gained per 50 residents per year by case study care home (CS1, CS2 and CS3) and intervention. Error bars represent the 95% percentile confidence intervals. Interventions: solar control window film (A1) or with external louvres and side fins (A2); increased thermal mass by stripping existing dry lining (B1) or by use of phase change materials (B2); active cooling by air conditioning (*a* = portable; *b* = wall-mounted) in communal spaces only (C1) or in communal spaces and bedrooms (C2); night time ventilation applied in communal spaces only (D1) or in communal spaces and bedrooms (D3+). Weather files: DSY = design summer year; TRY = test reference year.

The health benefits of interventions were greatest in CS1, followed by CS3 and CS2, respectively (figure A10). Across all interventions, the annual mean deaths averted per 50 residents ranged from 0.21–0.35 for CS1, 0.06–0.24 for CS2 and 0.00–0.21 for CS3. Similarly, the annual mean QALYs gained ranged from 0.08–0.65, 0.02–0.44 and 0.00–0.39 for CS1, CS2 and CS3, respectively. Intervention B2D3+ generally produced the most deaths averted, this was most evident in CS2 where deaths averted were around 50% more than the next most effective intervention.

Interventions producing a positive net monetary benefit were, for CS1: all interventions except for C2b, with B1D3+ achieving the highest net monetary benefit (£27 000); for CS2: D3+ (£1100), B2D3+ (£540), D1 (£530), C1a (£320), B1D3+ (£260) and A1D1 (£140); and for CS3: D3+ (£5200), B2D3+ (£3800), B1D3+ (£3200), A1D1 (£2800), C1a (£1100), A1 (£620), A2D1 (£230) and D1 (£140) (table A1).

### 3.4. Cost-effectiveness of building interventions

**Figure 1.**
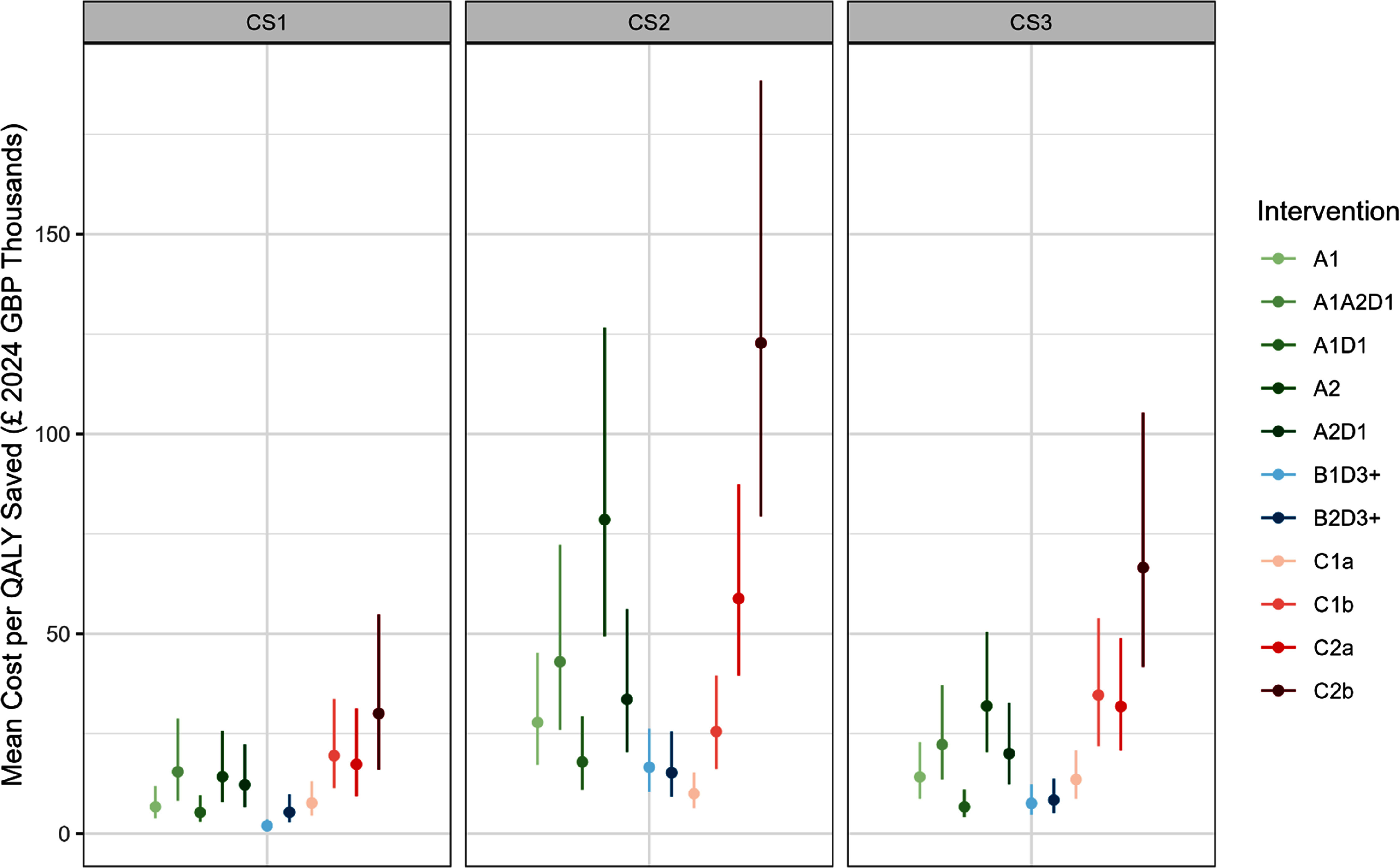
Mean cost per quality-adjusted life year (QALY) saved by intervention by care home (CS1, CS2 and CS3). Error bars represent the 95% percentile confidence intervals. Interventions: control window film (A1) or with external louvres and side fins (A2); increased thermal mass by stripping existing dry lining (B1) or by use of phase change materials (B2); active cooling by air conditioning (*a* = portable; *b* = wall-mounted) in communal spaces only (C1) or in communal spaces and bedrooms (C2); night time ventilation applied in communal spaces only (D1) or in communal spaces and bedrooms (D3+).

Under our pair-wise comparison with a common baseline approach, interventions were most cost-effective in CS1, where the mean cost per QALY saved ranged from around £2000–30 000 (figure 1). This was followed by CS3 and CS2, with ranges of £7000–67 000 and £10 000–123 000, respectively. In general, the least cost-effective interventions were C2b (£30 000–123 000) and A2 (£14 000–79 000), while the most cost-effective were B1D3+ and B2D3+ (£2000–17 000) and C1a (£8000–14 000).

In our fully incremental analysis, the most cost-effective interventions in CS1, CS2 and CS3 were B1D3+ (£1800 per QALY), C1a (£9500 per QALY) and A1D1 (£6300 per QALY), respectively (table 4). Interventions that were consistently dominated across all care homes were: A1A2D1, A2, A2D1, C1b and C2b.

**Table 4.** Fully incremental cost-effectiveness analysis. Interventions: solar control window film (A1) or with external louvres and side fins (A2); increased thermal mass by stripping existing dry lining (B1) or by use of phase change materials (B2); active cooling by air conditioning (*a* = portable; *b* = wall-mounted) in communal spaces only (C1) or in communal spaces and bedrooms (C2); night time ventilation applied in communal spaces only (D1) or in communal spaces and bedrooms (D3+). QALY = quality-adjusted life year; ICER = incremental cost-effectiveness ratio.

Care home	Intervention	Mean cost (£ 2024 GBP)	Mean benefit (QALYs)	ICER (£ per QALY)
CS1	B1D3+	2621	1.470	1783
	A1	5646	0.907	D
	A1D1	5646	1.157	D
	B2D3+	7257	1.497	4848
	C1a	8703	1.223	D
	A2	13 958	1.059	D
	A2D1	13 958	1.243	D
	A1A2D1	19 184	1.358	D
	C1b	22 160	1.223	D
	C2a	23 347	1.475	D
	C2b	40 438	1.475	D

CS2	C1a	295	0.031	9521
	C1b	753	0.031	D
	A1	788	0.030	D
	A1D1	788	0.046	ED
	B1D3+	943	0.060	15 719
	B2D3+	1380	0.096	14 363
	A2	1728	0.023	D
	A2D1	1728	0.055	D
	C2a	1980	0.035	D
	A1A2D1	2506	0.062	D
	C2b	4129	0.035	D

CS3	A1	1281	0.095	13 461
	A1D1	1281	0.202	6335
	B1D3+	1785	0.249	7161
	C1a	1981	0.153	D
	B2D3+	2466	0.311	7920
	A2	4012	0.132	D
	A2D1	4012	0.212	D
	C1b	5067	0.153	D
	A1A2D1	5481	0.261	D
	C2a	6968	0.230	D
	C2b	14 565	0.230	D

### 3.5. Sensitivity analysis

**Figure 2.**
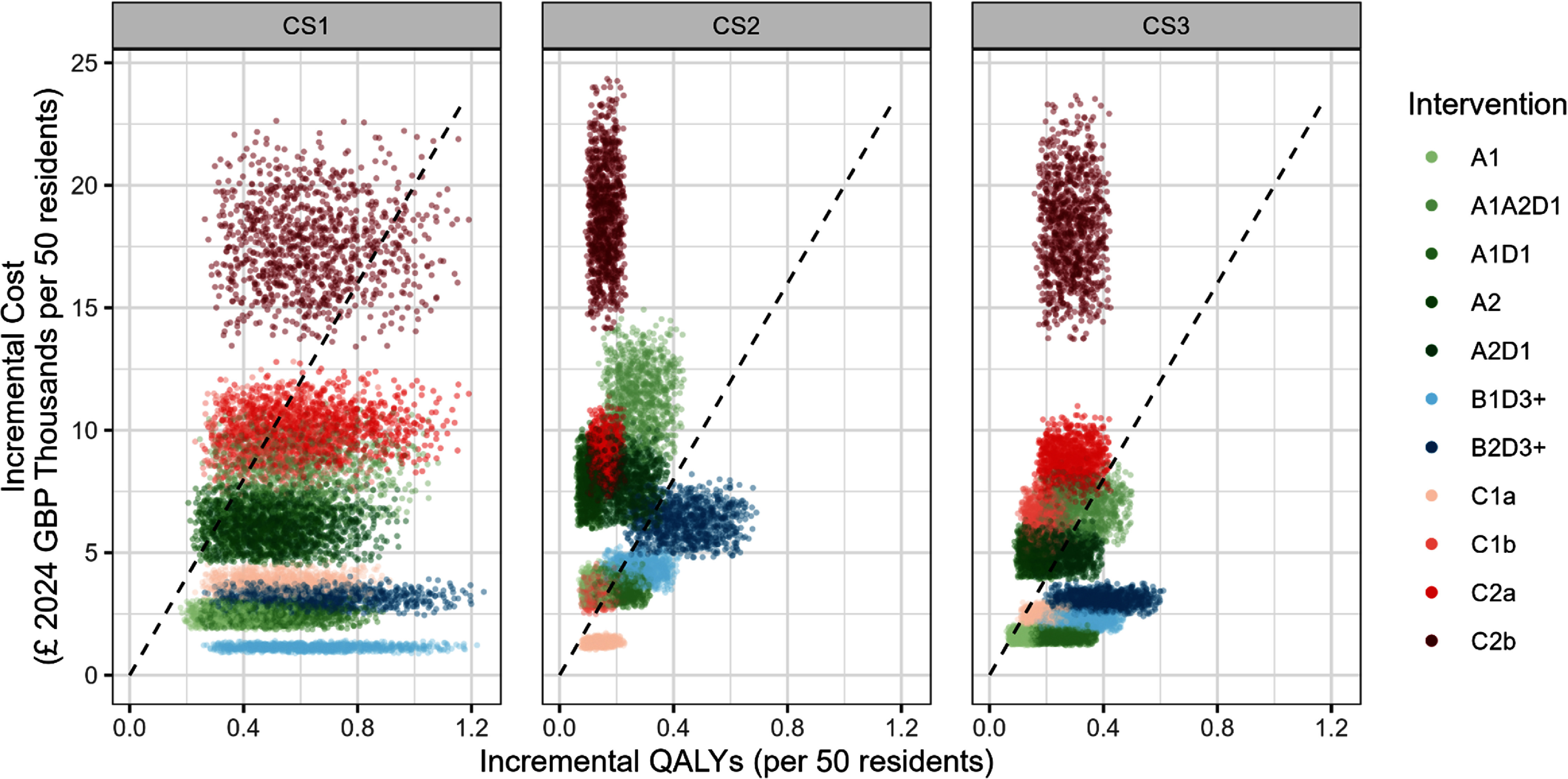
Cost-effectiveness plane for interventions in care homes (CS1, CS2 and CS3). Dashed line = £20 000 per quality-adjusted life year (QALY) threshold. Interventions: solar control window film (A1) or with external louvres and side fins (A2); increased thermal mass by stripping existing dry lining (B1) or by use of phase change materials (B2); active cooling by air conditioning (*a* = portable; *b* = wall-mounted) in communal spaces only (C1) or in communal spaces and bedrooms (C2); night time ventilation applied in communal spaces only (D1) or in communal spaces and bedrooms (D3+).

**Figure 3.**
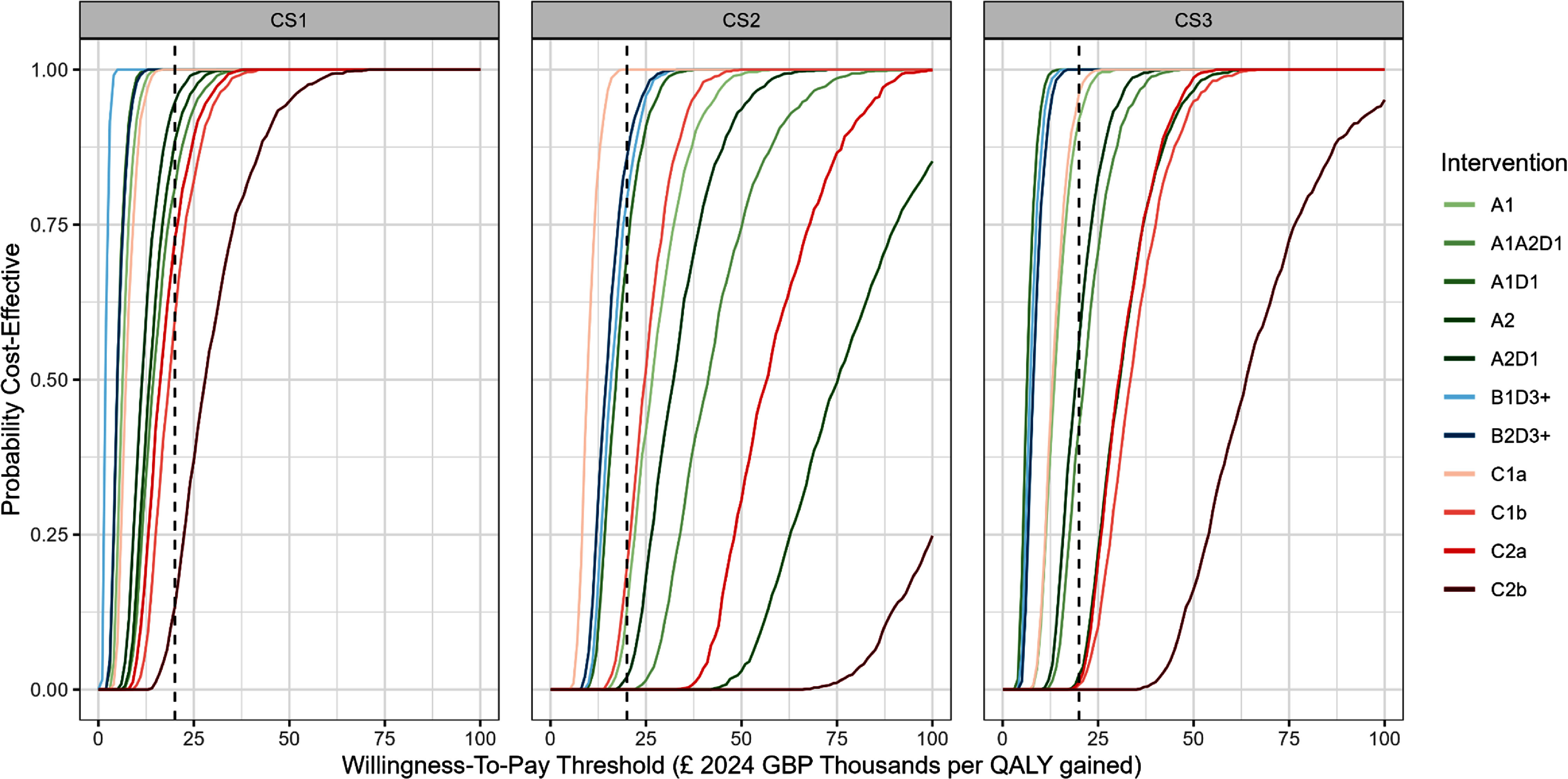
Cost-effectiveness acceptability curves for interventions in care homes (CS1, CS2 and CS3). Dashed line = £20 000 per quality-adjusted life year (QALY) threshold. Interventions: solar control window film (A1) or with external louvres and side fins (A2); increased thermal mass by stripping existing dry lining (B1) or by use of phase change materials (B2); active cooling by air conditioning (*a* = portable; *b* = wall-mounted) in communal spaces only (C1) or in communal spaces and bedrooms (C2); night time ventilation applied in communal spaces only (D1) or in communal spaces and bedrooms (D3+).

Under our pair-wise comparison with a common baseline approach, probabilistic sensitivity analysis showed that, for all care homes, there were several interventions that demonstrated cost-effectiveness at the £20 000 per QALY threshold (figures 2 and 3). In CS1, all interventions, except for C2b, appeared to be cost-effective at this threshold. By contrast, for CS2 and CS3, interventions that were more than 70% likely to be cost-effective at this threshold included, for CS2: C1a, B2D3+, B1D3+ and A1D1; and for CS3: A1D1, B1D3+, B2D3+, C1a and A1.

**Figure A11.**
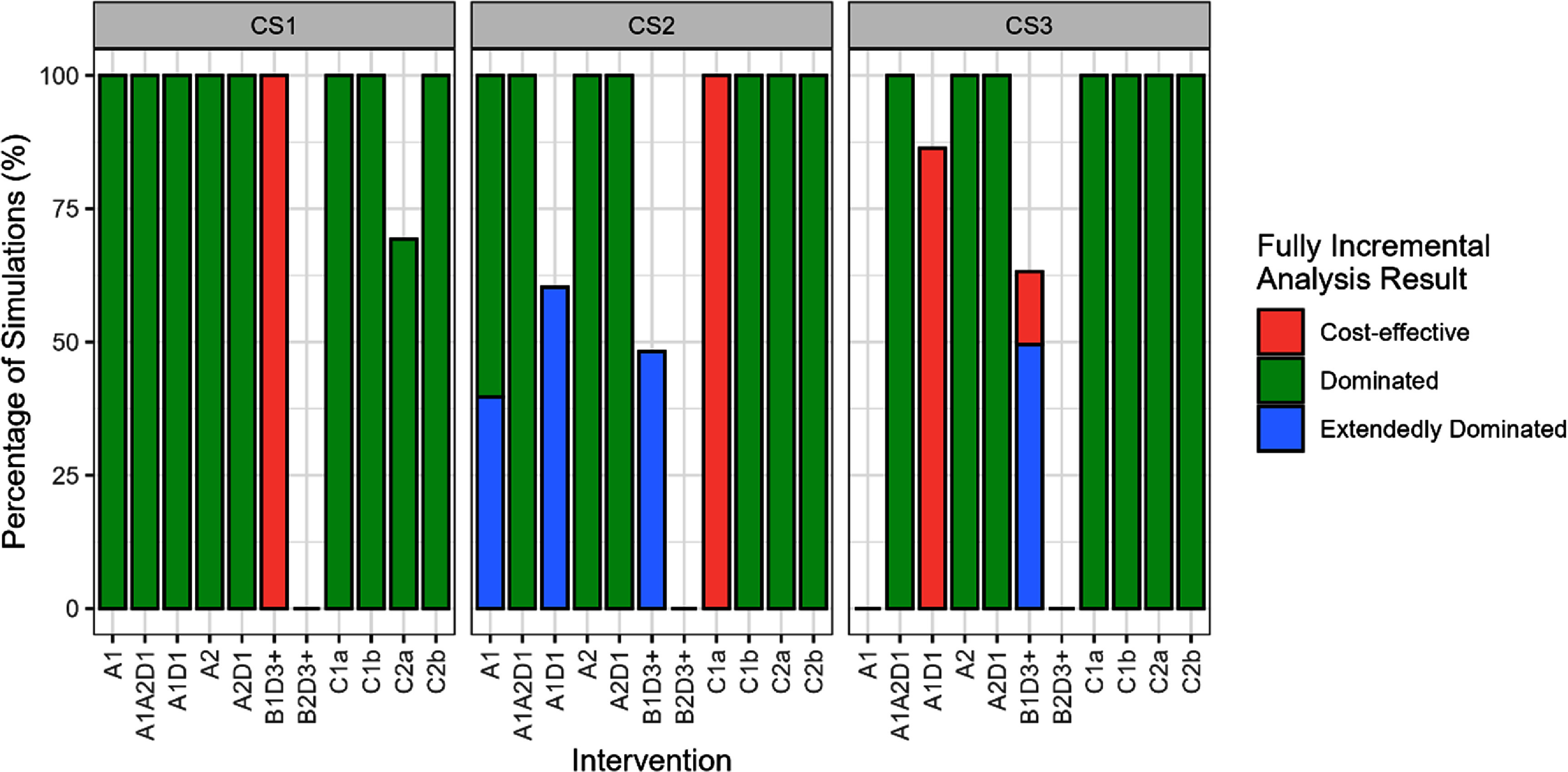
Results from our probabilistic sensitivity analysis using a fully incremental approach by case study care home (CS1, CS2 and CS3) and intervention. Interventions: solar control window film (A1) or with external louvres and side fins (A2); increased thermal mass by stripping existing dry lining (B1) or by use of phase change materials (B2); active cooling by air conditioning (*a* = portable; *b* = wall-mounted) in communal spaces only (C1) or in communal spaces and bedrooms (C2).

In our fully incremental approach, probabilistic sensitivity analysis showed that B1D3+ and C1a were always cost-effective in CS1 and CS2, respectively (figure A11). Whereas in CS3, A1D1 and B1D3+ had an 86% and 14% chance of being cost-effective, respectively. All other interventions, across all care homes, had zero chance of being cost-effective.

**Figure A12.**
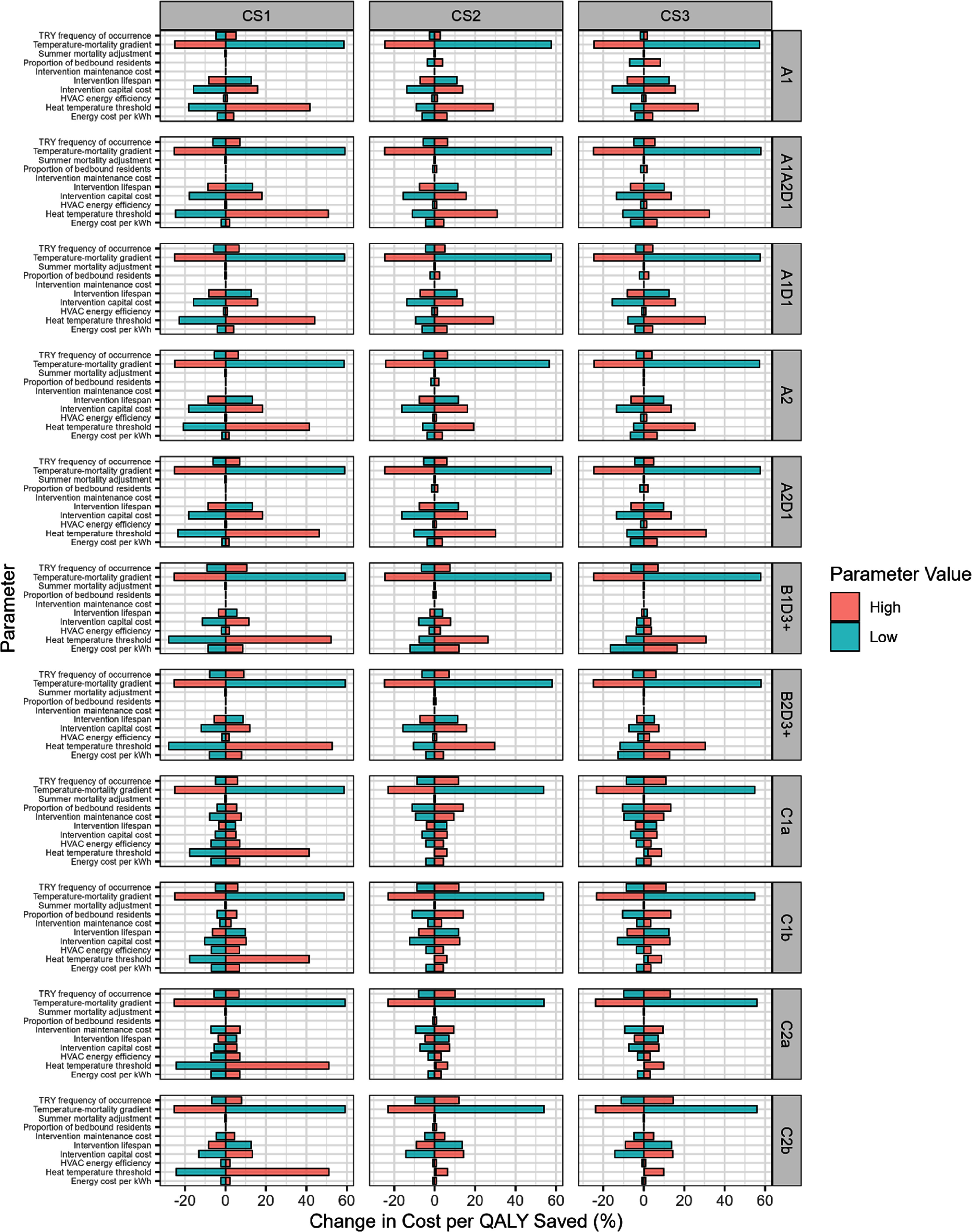
One-way sensitivity analysis of model parameters by case study care home (CS1, CS2 and CS3) and intervention. High and low parameter values are the minimum and maximum values of their assumed distributions detailed in table 3. Interventions: solar control window film (A1) or with external louvres and side fins (A2); increased thermal mass by stripping existing dry lining (B1) or by use of phase change materials (B2); active cooling by air conditioning (*a* = portable; *b* = wall-mounted) in communal spaces only (C1) or in communal spaces and bedrooms (C2).

One-way sensitivity analysis of model parameters showed that the cost-effectiveness of interventions was, in general, most sensitive to the temperature-mortality gradient and the heat temperature threshold (figure A12). The change in cost per QALY saved at high and low values of the temperature-mortality gradient was relatively consistent across all interventions and care homes, ranging from around −25% to 60%, respectively. Whereas at high and low values of the heat temperature threshold, changes ranged from around −30% to 50%, respectively, with large variation between care homes and interventions.

**Figure A13.**
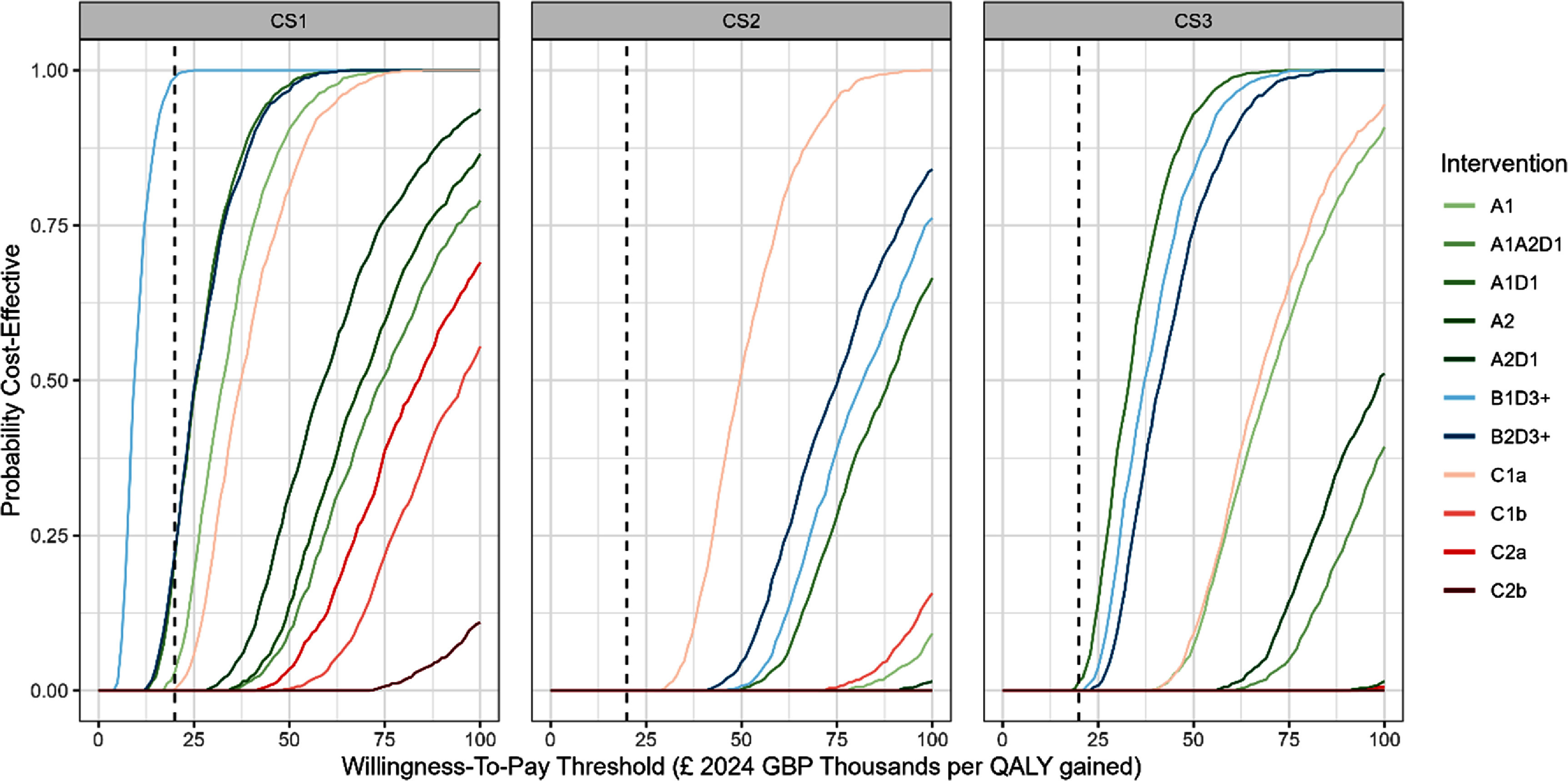
Cost-effectiveness acceptability curves for interventions in care homes (CS1, CS2 and CS3) under the conservative assumption that those who die from heat have a six-month life expectancy, regardless of age. Dashed line = £20 000 per quality-adjusted life year (QALY) threshold. Interventions: solar control window film (A1) or with external louvres and side fins (A2); increased thermal mass by stripping existing dry lining (B1) or by use of phase change materials (B2); active cooling by air conditioning (*a* = portable; *b* = wall-mounted) in communal spaces only (C1) or in communal spaces and bedrooms (C2).

Under the assumption that those who die of heat have a life expectancy of six-months, we found only one intervention, in one care home, to be cost-effective: increased thermal mass combined with window and door opening rules in CS1 (figure A13).

## 4. Discussion

This paper builds on previous research that explored methods to assess the mortality benefit of interventions to reduce heat risks in care homes among residents (Ibbetson *et al* 2021). Specifically, we conducted a health cost-benefit analysis which compares deliberately contrasting interventions across three case study care homes of differing size and building design to help identify candidate interventions for the cost-effective prevention of heat-related mortality.

Our results suggest that there are several interventions that may provide cost-effective prevention of heat-related mortality in care homes. In most cases, the cost-effectiveness of each intervention is determined by the building characteristics of the care home. Under our pair-wise comparison with a common comparator approach, interventions that appeared to be cost-effective at the £20 000 per QALY threshold in all three care homes were shading combined with window and door opening rules (A1D1), increased thermal mass combined with window and door opening rules (B1D3+ and B2D3+) and active cooling in lounges using portable air-conditioners (C1a). The only intervention that appeared to be universally not cost-effective was wall-mounted air-conditioning to selected communal spaces and bedrooms (C2b), mainly due to its high capital costs.

Our results also indicate that behavioural interventions, such as window and door opening, if fully adhered to, may be among the most effective interventions at reducing exposure to heat-related risks in some care homes. It is perhaps unexpected to see these types of interventions outperform air-conditioning. This result is likely due to our chosen set point (26 °C) for the air-conditioning and our method to calculate health impact which relies on the outdoor temperature-mortality function. For example, if the indoor temperature is below the set point, but the outdoor temperature is above the heat mortality threshold, the air-conditioning will not be operating despite there being, according to our method, some risk of heat-related mortality. This demonstrates the need for an indoor temperature-mortality function.

To our knowledge, only one study has previously investigated the cost-effectiveness of heat interventions in care homes. This study explored the health monetary benefit of shading with external louvres and side fins (A2) in a care home of 50 residents (Ibbetson *et al* 2021). Their results suggest that, under the assumption that those who die of heat have a life expectancy of six-months, this intervention could be justified at a willingness-to-pay threshold of £30 000 per QALY if its capital costs were around £40 000. Our results for the same intervention are in line with their estimates of effectiveness in terms of life years gained. However, our results indicate that this intervention is potentially less cost-effective than their estimate for several reasons. First, their cost estimate of this intervention was around 46% less than our estimate. This above inflation increase in cost of the bespoke shading device may reflect differences between suppliers engaged at different time periods (pre- and post-pandemic), the high variability typically associated with bespoke fabrication costs and the differences in the building sizes on which costings were based. Second, we adjusted for quality of life using age-specific population norms, where they made no adjustment. Third, we used a willingness-to-pay threshold of £20 000 per QALY whereas they used £30 000 per QALY.

Our study has several strengths. First, we assessed interventions across three care homes with different building characteristics. This allowed us to assess the relative performance of interventions across care homes of different design. Second, we evaluated a wide range of contrasting interventions, providing more options to care home managers and policymakers on potentially cost-effective options. Third, we rigorously assessed model uncertainty through a deterministic and probabilistic sensitivity analysis.

Our study also has several limitations. First, a large degree of uncertainty surrounds our assumptions over loss of life expectancy. Studies which quantify the effect of heat on mortality are typically based on daily time-series designs where it is not possible to establish the loss of life expectancy from heat-related mortality (Armstrong *et al* 2017, Rehill *et al* 2015). The extent of life-shortening is likely to vary by population and age due to differences in causes of heat-related mortality (Hajat *et al* 2005). Care home residents, who are often in poor health and near the end of their life, may only lose months or weeks of life. We explored this uncertainty in a sensitivity analysis which made the conservative assumption that those who die of heat have a life expectancy of six-months. Under this assumption we found only one intervention, in one care home, to be cost-effective.

Second, by applying population level estimates of relative risk for the period 1993–2003 to our case study care homes, we assume that they represent the average care home in England and Wales during this period. However, each care home is likely to have a unique temperature-mortality function which is based on its building features and the characteristics of its population. In addition, the association between heat and mortality may have changed since that period. We explored uncertainty relating to the heat-mortality function in our sensitivity analysis which demonstrated that the cost-effectiveness of interventions was highly sensitive to the temperature-mortality gradient. Future research could address this limitation by producing a more recent estimate of the temperature-mortality function for care home residents and by exploring the influence of the building and population characteristics of care homes on the temperature-mortality function.

Third, our use of age-specific population norms for care home residents probably overestimates their quality of life, biasing our cost-effectiveness estimates downwards and weakening the case for the £20 000 per QALY threshold. On the one hand, care home residents are likely to have poorer physical health than individuals of the same age in the general population (O’Neill *et al* 2020). On the other hand, our assessment of health benefit is based purely on reduction in heat-related mortality, so our estimates do not include the potential quality of life benefits from reduced heat morbidity (Ebi *et al* 2021).

Finally, we acknowledge that uncertainties in building modelling inputs and outputs also exist despite having previously validated the models used in this study (Oikonomou *et al* 2025). Thus, the findings from this study are conditional on the specified model inputs which were informed by the best-available information.

## 5. Conclusion

This study provides new evidence for an increasing focus on interventions to reduce the health effects of heat in care homes. We have demonstrated that several interventions can be cost-effective in a range of care home types. These interventions include shading and increased thermal mass, in combination with window and door opening rules, and air-conditioning in communal areas. However, the most suitable intervention is likely to be unique to the characteristics of the specific care home. Decision-makers should therefore consider a variety of interventions when assessing their options. Future research could strengthen guidance for decision-makers by examining how different care home characteristics influence the relative cost-effectiveness of interventions, thereby improving our ability to provide targeted recommendations on which interventions are likely to be most cost-effective in their setting.

## Data Availability

The data cannot be made publicly available upon publication because no suitable repository exists for hosting data in this field of study. The data that support the findings of this study are available upon reasonable request from the authors.

## Appendix

**Table A1.** Net monetary benefit of interventions for a willingness-to-pay threshold of £20 000 per quality-adjusted life year (QALY). Costs are reported in £ 2024 GBP. Interventions: solar control window film (A1) or with external louvres and side fins (A2); increased thermal mass by stripping existing dry lining (B1) or by use of phase change materials (B2); active cooling by air conditioning (*a* = portable; *b* = wall-mounted) in communal spaces only (C1) or in communal spaces and bedrooms (C2); night time ventilation applied in communal spaces only (D1) or in communal spaces and bedrooms (D3+).

Care home	Intervention	Cost (£)	Benefit (QALYs)	Net monetary benefit (£)
CS1	A1	5646	0.907	12 496
	A1A2D1	19 184	1.358	7973
	A1D1	5646	1.157	17 488
	A2	13 958	1.059	7225
	A2D1	13 958	1.243	10 900
	B1D3+	2621	1.470	26 771
	B2D3+	7257	1.497	22 682
	C1a	8703	1.223	15 751
	C1b	22 160	1.223	2294
	C2a	23 347	1.475	6154
	C2b	40 438	1.475	−10 936
	D1	0	0.919	18 376
	D3+	0	1.471	29 411

CS2	A1	788	0.030	−189
	A1A2D1	2506	0.062	−1269
	A1D1	788	0.046	142
	A2	1728	0.023	−1266
	A2D1	1728	0.055	−638
	B1D3+	943	0.060	257
	B2D3+	1380	0.096	542
	C1a	295	0.031	324
	C1b	753	0.031	−134
	C2a	1980	0.035	−1277
	C2b	4129	0.035	−3427
	D1	0	0.027	532
	D3+	0	0.056	1122

CS3	A1	1281	0.095	622
	A1A2D1	5481	0.261	−258
	A1D1	1281	0.202	2763
	A2	4012	0.132	−1368
	A2D1	4012	0.212	229
	B1D3+	1785	0.249	3200
	B2D3+	2466	0.311	3761
	C1a	1981	0.153	1085
	C1b	5067	0.153	−2001
	C2a	6968	0.230	−2374
	C2b	14 565	0.230	−9971
	D1	0	0.007	136
	D3+	0	0.261	5225
